# A multigenotype maize silk expression atlas reveals how exposure‐related stresses are mitigated following emergence from husk leaves

**DOI:** 10.1002/tpg2.20040

**Published:** 2020-10-14

**Authors:** Colton McNinch, Keting Chen, Tesia Dennison, Miriam Lopez, Marna D. Yandeau‐Nelson, Nick Lauter

**Affiliations:** ^1^ Molecular, Cellular, and Developmental Biology Graduate Program Iowa State Univ. Ames IA 50011 USA; ^2^ Bioinformatics & Computational Biology Graduate Program Iowa State Univ. Ames IA 50011 USA; ^3^ Genetics & Genomics Graduate Program Iowa State Univ. Ames IA 50011 USA; ^4^ USDA‐ARS Corn Insects and Crop Genetics Research Unit Iowa State Univ. Ames IA 50011 USA; ^5^ Department of Genetics, Development and Cell Biology Iowa State Univ. Ames IA 50011 USA

## Abstract

The extraordinarily long stigmatic silks of corn (*Zea mays* L.) are critical for grain production but the biology of their growth and emergence from husk leaves has remained underexplored. Accordingly, gene expression was assayed for inbreds ‘B73’ and ‘Mo17’ across five contiguous silk sections. Half of the maize genes (∼20,000) are expressed in silks, mostly in spatiotemporally dynamic patterns. In particular, emergence triggers strong differential expression of ∼1,500 genes collectively enriched for gene ontology terms associated with abiotic and biotic stress responses, hormone signaling, cell–cell communication, and defense metabolism. Further, a meta‐analysis of published maize transcriptomic studies on seedling stress showed that silk emergence elicits an upregulated transcriptomic response that overlaps strongly with both abiotic and biotic stress responses. Although the two inbreds revealed similar silk transcriptomic programs overall, genotypic expression differences were observed for 5,643 B73–Mo17 syntenic gene pairs and collectively account for >50% of genome‐wide expression variance. Coexpression clusters, including many based on genotypic divergence, were identified and interrogated via ontology‐term enrichment analyses to generate biological hypotheses for future research. Ultimately, dissecting how gene expression changes along the length of silks and between husk‐encased and emerged states offers testable models for silk development and plant response to environmental stresses.

**Abbreviations**
Bx: benzoxazinoid
CRP: cysteine‐rich peptide
DEG: differentially expressed gene
GCN: gene coexpression network
GO: gene ontology
JA: jasmonic acid
LOX: lipoxygenase
*Pin1*, *‐2*, and *‐10*
: *PIN‐FORMED1*, *PIN‐FORMED2*, and *PIN‐FORMED10*
RPKM: transcript‐length‐normalized reads per million reads
SA: salicylic acid
TF: transcription factor
TOM: topographical overlap matrix.

## 1 INTRODUCTION

As stigmatic organs, maize silks are required for ovule fertilization, which occurs ∼7 × 10^15^ times annually as part of global corn grain production. Maize yields depend on the successful emergence of silks from the husk leaves that protectively encase the ear (Bolaños & Edmeades, 1996) and on the sustained viability of silks for pollen reception under harsh environmental conditions after emergence (Bassetti & Westgate, 1993). To overcome the first of these challenges, silks grow more than 1 cm d^–1^ for 10 to 15 d until emergence from the husk leaves, after which cellular elongation continues at lesser rates (Fuad‐Hassan, Tardieu, & Turc, 2008). To continue to grow and to be pollen‐receptive upon emergence, nutrient levels and osmotic pressures must be maintained (Westgate & Boyer, 1985). This, in turn, requires silks to use physical and biochemical mechanisms to guard against the water deficits, pathogens, and pests that are typical of midsummer (Ortega Corona, 1987; Pechanova & Pechan, 2015). Indeed, silks exhibit a high level of phenotypic variability in emergence rates across diverse germplasm under drought (Bolaños & Edmeades, 1996), as well as differences in susceptibility to different pathogens (Lübberstedt, Klein, & Melchinger, 1998) and pests (Abel, Wilson, Wiseman, White, & Davis, 2000; Lopez et al., 2019). Moreover, the composition of many specialized metabolites varies among cultivars, including maysin (Byrne et al., 1996; Szalma, Buckler, Snook, & McMullen, 2005) and cuticular lipids (Dennison et al., 2019; Loneman et al., 2017; Perera et al., 2010).

The emergence of plant tissues, such as during seedling emergence from the ground or monocot leaf emergence from a whorl, introduces microenvironmental changes and therefore new stresses. Emergence responses can be difficult to study because they are often indiscrete and accompanied by confounding changes. For example, when maize leaves emerge from their protective leaf whorls, they do so gradually while still undergoing morphogenesis and photosynthetic differentiation from sink to source tissue (Li et al., 2010; Pick et al., 2011). By contrast, maize silks experience a more abrupt transition at their constricted point of emergence after having completed nearly all stages of morphogenesis (Fuad‐Hassan et al., 2008), offering a tractable model system in which to study emergence and stress responses.

The molecular reprogramming of silks as they elongate and emerge has remained underexplored, despite the inclusion of silks (Supplemental Table S1) as a biosample type in expression atlases (Stelpflug et al., 2016; Walley et al., 2016; Zhou, Hirsch, Briggs, & Springer, 2019) and as part of more focused transcriptomic studies (Moran Lauter, Muszynski, Huffman, & Scott, 2017; Morohashi et al., 2012; Xu et al., 2012; Xu, Wang, Chen, Sun, & Zhang, 2013). To uncover the genetic and environmental variation in spatiotemporal gene expression, the husk‐encased and emerged portions of silks from inbreds B73 and Mo17 were subsampled along a proximal–distal gradient at 3 d following emergence from husk leaves. We chose the inbred lines B73 and Mo17 because they are foundational representatives of two major heterotic patterns in modern maize hybrid breeding (Troyer, 1999) and therefore exhibit considerable genetic variation (Beló et al., 2010; Lai et al., 2010). Moreover, these two inbreds are particularly well suited for comparisons of gene expression because of the availability of long‐read genome sequence assemblies that have comparably high qualities (Jiao et al., 2017; Sun et al., 2018).

**Core Ideas**

- Stigmatic silks of maize are unique, important to agriculture, and underexplored.
- Gene expression is dynamic along the silk length and between genotypes.
- Silk emergence from the husk leaves elicits abiotic and biotic stress responses.
- A consensus RNA‐Seq method to leverage >1 genome assembly is introduced.
- Genotypic comparisons highlight both conserved and variable properties of silks.
- Corn silks and leaves share morphogenesis programs but develop in distinct ways.

In this study, multigenotype spatiotemporal approach permitted the investigation of interesting and largely unaddressed areas of silk biology, including (a) how silk responds to emergence into the external environment, (b) what genetically conserved and distinct expression patterns exist along the lengths of silks, and (c) where gene expression related to cell division, expansion, and differentiation occurs along the developmental gradient. To assess the functional relevance of the observed expression dynamics, Gene Ontology (GO) and annotation mining, as well as meta‐analysis of prior studies and coexpression network analysis were used, revealing important roles of gene expression in silk development, metabolism, physiology, and abiotic and biotic defense.

## 2 MATERIALS AND METHODS

### 2.1 Plant materials, experimental design, and data acquisition

**FIGURE 1:**
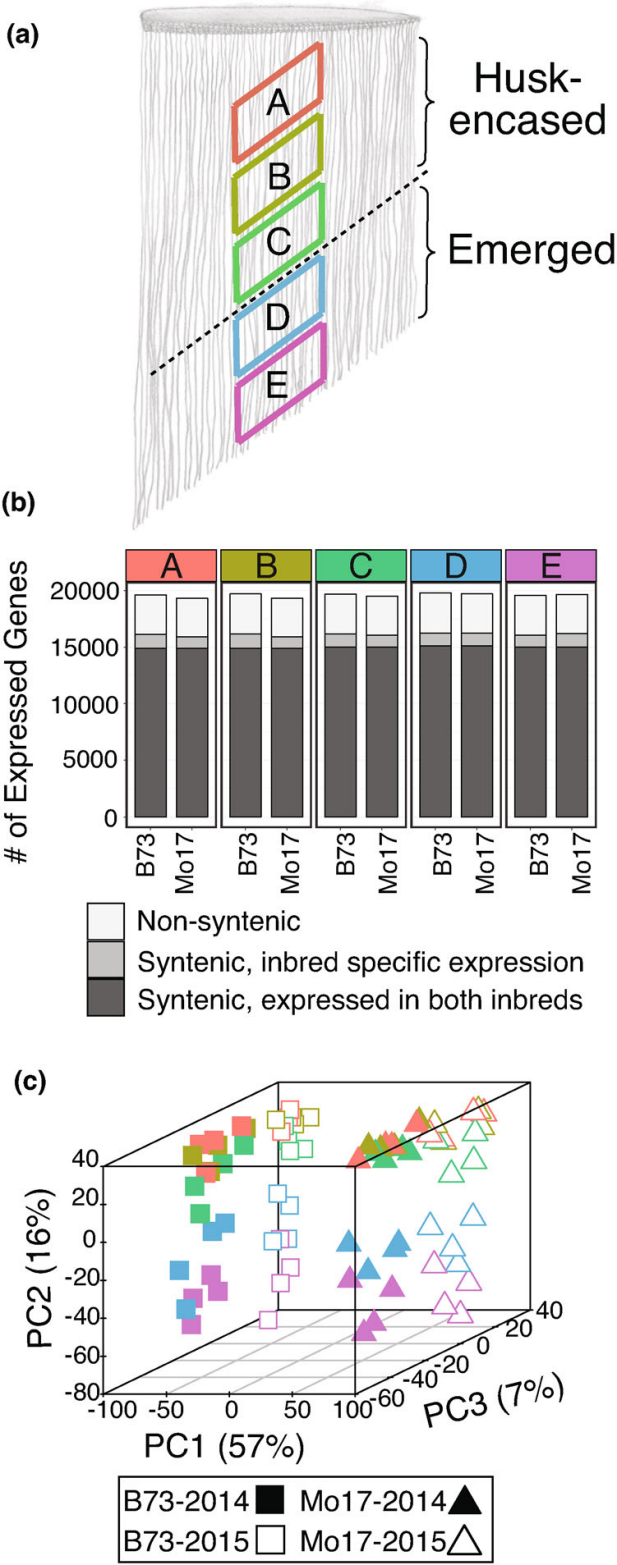
Global gene expression along the silk length for maize inbreds B73 and Mo17. (a) This “curtain of silks” diagram depicts the the penultimate step for sampling three husk‐encased and two emerged silk sections from ears collected 3 d after silk emergence. Each ear was sliced longitudinally, facilitating removal of the husk leaves, which, in turn, allowed the silks belonging to the medial third of the ear to be separated and sliced into five samples. The emergence boundary is diagonal in the curtain because the longest silks belong to the base of the ear. This diagonal does not intersect the ear tip because the husk leaves are longer than the immature ear they encase. (b) Total number of expressed genes (average transcript‐length‐normalized reads per million reads > 1) in each section are displayed for both inbreds, with bar shades indicating syntenic expression states. (c) Principal Component (PC) analysis demonstrating the associations among the 80 silk samples with syntenic gene expression (*n* = 24,928 gene pairs). The percentage of variance explained by each PC is shown. Symbol colors represent silk section

Public accessions of B73 (PI 550473) and Mo17 (Ames 29979) were grown to maturity in Webster clay‐loam soil (fine‐loamy, mixed, superactive, mesic Typic Endoaquolls) in Boone County, IA, at 0.76‐m row spacing and 0.22‐m plant spacing. Staggered plantings in May in both years (2014 and 2015) allowed synchronous tissue harvests in midsummer. Ears were covered by shoot bags prior to silk emergence and visited daily to mark silk emergence. At 3 d after silk emergence, whole ears from ideotypic plants were picked at midday and held at ambient conditions until silk sampling. In both years, four bioreplicates, each consisting of two pooled ears, were sampled per inbred. For each pool, silks were dissected into five sections as follows. First, the husk‐encased ears were sliced longitudinally, facilitating removal of the husks. Second, each half ear was held horizontally in the air so that the silks dangled, allowing removal of the distal and proximal quarters of the ear. Third, the medial portion of the ear was laid on a cutting board and cut into Sections A, B, C, D, and E (Figure 1). The initial cut was made to separate encased silks from emerged ones, which is demarcated by a kink in the silks where the husk encasement pinched the plume of emerging silks into a tight channel. The second cut (parallel to the first) was made to divide the emerged portion into Sections D and E of equal length. Finally, two additional parallel cuts were made to divide the encased portion into Sections A, B, and C of equal length. This method is designed to be extensible across corn genotypes with varying lateral branch morphologies but does allow the lengths of encased and emerged sections to differ even within a genotype. Specifically, the encased length depends on the length of the husk encasement, whereas the emerged length depends on the amount of silk elongation during the 3‐d period between initial emergence and sampling. In this study, the five silk sections were approximately the same height for all ears involved because both B73 and Mo17 have ∼17‐cm‐tall husk encasements and produce ∼9 cm of emerged silks in the first 3 d after silking. Although all dissections on a single day were performed within a few minutes, sampling order was alternated by genotype to alleviate potential temporal sampling effects. In total, 80 samples were collected (2 yr × two inbreds × four bioreplicates × five silk sections).

For each biosample, total RNA was isolated from cryo‐ground tissue by buffered thiocyanate salts and phenol‐chloroform extractions as previously described (Moscou, Lauter, Steffenson, & Wise, 2011). mRNA purification, library construction, and barcoding steps were performed by the DNA Facility staff at Iowa State University with Illumina TruSeq Kits (Illumina, San Diego, CA). The 50‐cycle sequence data were acquired from eight lanes of Illumina HiSeq 3000 in high output mode, with each lane containing 10 biosamples comprising an A to E set from each genotype. The sequence data and the detailed metadata can be accessed through the NCBI Sequence Read Archive, accession No. PRJNA545496.

### 2.2 RNA‐Sequencing analysis

RNA‐Sequencing reads were preprocessed to remove adapter sequences and examined with FastQC (http://www.bioinformatics.babraham.ac.uk/projects/fastqc, accessed 16 July 2020), revealing an average read length of 49 bp and a median read depth of 34.4 million reads per biosample. The HISAT2 alignment program (Kim, Langmead, & Salzberg, 2015) was used to align the reads from B73 and Mo17 samples to the B73 (Jiao et al., 2017) and Mo17 (Sun et al., 2018) reference genomes under the default settings. The percentage of reads per sample with unique alignments and with no alignments was then used as a criterion to evaluate which reference genome was best suited for B73 and Mo17 samples. The B73 samples had more uniquely aligned and fewer unaligned reads when aligned to the B73 genome. Similarly, Mo17 samples had more uniquely aligned and fewer unaligned reads when aligned to the Mo17 genome (Supplemental Figure S1). As such, the B73 and Mo17 reference genomes were used for aligning reads from B73 and Mo17 samples respectively. Next, transcripts were assembled and quantified on a reads per kb of transcript per million mapped reads (RPKM) and absolute read count basis with the StringTie transcript assembler (Pertea et al., 2015). In this last step, the –M flag was set to 0.5 to exclude loci for which more than 50% of reads mapped to multiple locations.

### 2.3 Identification of distinct pairs of syntenic genes

The SynMap comparative genomics tool (Lyons, Pedersen, Kane, & Freeling, 2008) was used to identify genes that were syntenic between the unmasked B73 and Mo17 reference genomes. SynMap was run with the MegaBlast algorithm set to an e‐value threshold of 0.001 and with the minimum number of aligned pairs at three genes. Syntenic blocks were merged by the Quota Align algorithm and with a coverage depth ratio of 1:1. All other SynMap parameters were set to the default values. Through this process, 25,057 B73 and 25,066 Mo17 genes were determined to possess synteny with one or more genes from the other genome (Supplemental Table S2). A distinct set of 24,928 syntenic gene pairs with 1‐to‐1 correspondence between the genomes was then identified and used in subsequent stages of this investigation.

### 2.4 Principal component analysis

The relationship among the transcriptome profiles of samples was examined via principal component analysis. This was achieved by the log_2_(RPKM + 1) normalized expression statistics of protein‐coding genes. Principal component analysis was undertaken on each inbred separately and in combination via the prcomp function in the R statistical software environment (R Core Team, 2018). Samples were plotted in a Cartesian coordinate system on the basis of loadings identified in the first (*x*‐axis), second (*y*‐axis), and third (*z*‐axis) (Figure 1c) principal components. Normal 95% confidence ellipses were projected onto all two‐dimensional plots to represent the multidimensional confidence intervals that encompass samples collected during the same growing year (i.e., 2014 or 2015) and silk region (i.e., husk‐encased or emerged) via the ggplot2 R package (Wickham, 2016).

### 2.5 Differential expression analysis

Differential expression analyses were performed with the DESeq2 R package (Love, Huber, & Anders, 2014) on absolute read counts per gene. Genes were considered to be differentially expressed if they had a false discovery rate‐adjusted p‐value less than 0.05 and absolute fold‐change differences of >2. Additionally, genes with effectively no expression (<5 reads) in most (>75%) of the samples in a given comparison were disregarded to ensure that stochastically expressed genes were depleted from differentially expressed gene (DEG) lists. Analyses between samples from the same inbred were made on read counts generated when that inbred's reference genome was used for read alignment and expression quantification. A consensus approach was taken for analyses between samples from different inbreds, wherein two separate analyses were completed with read counts generated by aligning all samples to both the B73 and Mo17 reference genomes. Genes differentially expressed in the same way (i.e., consistently higher in the same inbred) in both analyses were considered as DEGs, thereby removing the potential for the choice of reference genome to impact the outcomes.

### 2.6 Gene ontology enrichment

Gene Ontology enrichment tests were performed on various gene lists with the topGO R package (Alexa, Rahnenführer, & Lengauer, 2006) and maize‐GAMER GO annotations (Wimalanathan, Friedberg, Andorf, & Lawrence‐Dill, 2018) created for protein‐coding genes of the B73 genome. TopGO's weight algorithm was used to identify enriched GO terms because it integrates GO graph topology through a gene‐weighting approach to minimize the interdependencies of related GO terms. Statistical significances of GO terms with 10 or more genes assigned to them were computed via Fisher's exact test. Gene Ontology terms with a *p*‐value less than 0.001 were considered as significantly enriched.

### 2.7 Identification of cysteine‐rich peptides

Genes encoding cysteine‐rich peptides (CRPs) were identified by first scanning the longest transcripts of all B73 and Mo17 gene models for membrane signal sequence domains with SignalP5.0 (Almagro Armenteros et al., 2019). Transcripts with a signal sequence domain were then scanned for a transmembrane helix domain with TMHMM Version 2.0 (Krogh, Larsson, von Heijne, & Sonnhammer, 2001) after removing their predicted amino‐terminal signal sequence domains. Transcripts possessing a signal sequence domain and at least one transmembrane helix domain were regarded as localized to the plasma membrane. Lastly, CRPs were identified by scanning the remaining signal sequence domain‐containing transcripts with plant CRP Hidden Markov Models (Silverstein et al., 2007) by HMMER3 (Eddy, 2011).

### 2.8 Overlap enrichment tests of DEG sets

We used the GeneOverlap R package (Shen & Sinai, 2018) to examine DEGs between husk‐encased and emerged silks for enrichments of differential expression under five abiotic (drought, heat, ultraviolet light, salt, and cold) and two biotic [jasmonic acid (JA) and salicylic acid (SA) treatment] stress‐related conditions. Fisher's exact test was used to identify the overlap (intersection) significance between up‐ and downregulated gene lists identified in this study and the up‐ and downregulated gene lists identified in previous studies that examined seedling transcriptomic responses to stress conditions (Supplemental Table S3; Makarevitch et al., 2015; Wang et al., 2017; Zhang, Lei, Lai, Zhao, & Song, 2018; Ziemann et al., 2018). Because DEGs between emerged and encased silks may be associated with the developmental aspects of the encased–emerged transition, negative controls for this analysis were extracted from DEG lists derived from developmental contrasts between samples of embryos (0 vs. 20 d after pollination), internodes (mature vs. immature), leaves (division zone vs. elongation zone), and primary roots (elongation zone vs. maturation zone), which could then be compared against the seedling stress DEG lists (Supplemental Table S3; Li et al., 2010; Walley et al., 2016).

### 2.9 Transcription factor family enrichment tests

Enrichments of transcription factor (TF) families for differential expression between husk‐encased and emerged silks were tested via Fisher's exact test. This was done by determining the significance of the overlap between gene members of a particular TF family and the list of DEGs between husk‐encased and emerged silks. The annotated B73 TFs (Yilmaz et al., 2009) were used in this analysis along with the GeneOverlap R package. The TF families were considered to be significantly enriched for differential expression if the *p*‐values of overlap significance were less than 0.01.

### 2.10 Coexpression analysis

Syntenic genes expressed (average RPKM > 1) in at least one silk section of B73 or Mo17 were used to construct the syntenic gene‐based coexpression network.

Log_2_‐normalized RPKM values of all 80 RNA‐Sequencing samples were used with B73 and Mo17 samples as determined by their normalized expression obtained when using the B73 and Mo17 reference genomes, respectively. As such, distinct gene pairs (i.e., a B73 and Mo17 gene model) represent each syntenic gene comprising the network. The Weighted Gene Co‐expression Network Analysis R package (Langfelder & Horvath, 2008) was used to construct a signed network by first creating an adjacency matrix derived through biweight midcorrelations and a soft power threshold of 16. Next, a Topographical Overlap Matrix (TOM) was created by the TOMsimilarity function with the default parameters. Hierarchical clustering with the hclust function was then performed on the TOM distance matrix (1 – TOM) to derive an initial set of coexpressed gene clusters. Clusters were further refined by the cutreeDynamic function with the deepSplit parameter set to two and a minimum cluster size of 30. Subsequently, highly similar clusters were merged by the mergeCloseModules function with the cutHeight parameter set to 0.15. To display the expression patterns characteristic of the clusters, average eigengene values were computed for each inbred × section combination without regard to growing year.

## 3 RESULTS

### 3.1 Spatiotemporal transcriptome profiling of maize silks from B73 and Mo17 inbreds

To define the spatiotemporal patterns of maize silk gene expression, we conducted a replicated (×4), multigenotype (×2), multienvironment (×2) RNA‐Sequencing experiment on five contiguous sections (×5) of unpollinated silk tissue at 3 d after silk emergence (Figure 1). More than 2.9 billion single‐end reads were sequenced across the samples and aligned to their respective B73 (Jiao et al., 2017) and Mo17 (Sun et al., 2018) reference genome assemblies. This alignment strategy substantially improved the mapping ability of the reads compared with when only a single genome was used as the reference (Supplemental Figure S1). For instance, use of the Mo17 genome for Mo17 read alignments led to, on average, 3.79 million (9.2% of total reads) fewer unaligned reads than when the B73 genome was used. Thus RPKM values were computed with the corresponding reference genome in each case. Examination of these data indicates that similar numbers of genes were expressed (average RPKM > 1) in the two inbreds in each of the five silk sections. In addition, most of these genes were syntenic and expressed in both B73 and Mo17 (Figure 1b). Of the 20,808 B73 and 20,710 Mo17 genes were expressed in at least one silk section, 18,511 and 18,381 were expressed in all sections, respectively. Genes not expressed in all sections were most often expressed in neighboring silk sections (Section Figure S2). Nonsyntenic genes exhibited no silk expression more often than would be expected by chance in Fisher's exact tests (*p* < 2.2 × 10^–16^ for both B73 and Mo17), suggesting that gene models with support in only one assembled genome are not as reliable as those identified by both. However, there are certainly true cases of presence–absence variation between these two inbreds, making these expression data useful for characterizing the presence variants.

The overall relationship between samples was determined via a principal component analysis that used the expression of all genes that were syntenic between the two inbreds (*n* = 24,928) as input. This analysis revealed tight grouping of biological replicates and distinct separation among samples collected from different inbreds, regions of the silk, and growing year (Figure 1c). Samples also shared higher similarity to other samples from the same husk encasement status (i.e., husk‐encased vs. emerged), a trend that was most apparent in individual principal component analysis plots for each inbred (Supplemental Figure S3). In analyses for both inbreds, the 95% multidimensional confidence intervals encompassed samples collected from the same silk encasement status (i.e., husk‐encased or emerged) and from the same growing year (i.e., 2014 or 2015).

### 3.2 Changes in gene expression and enrichment of GO terms along the silk length

**FIGURE 2:**
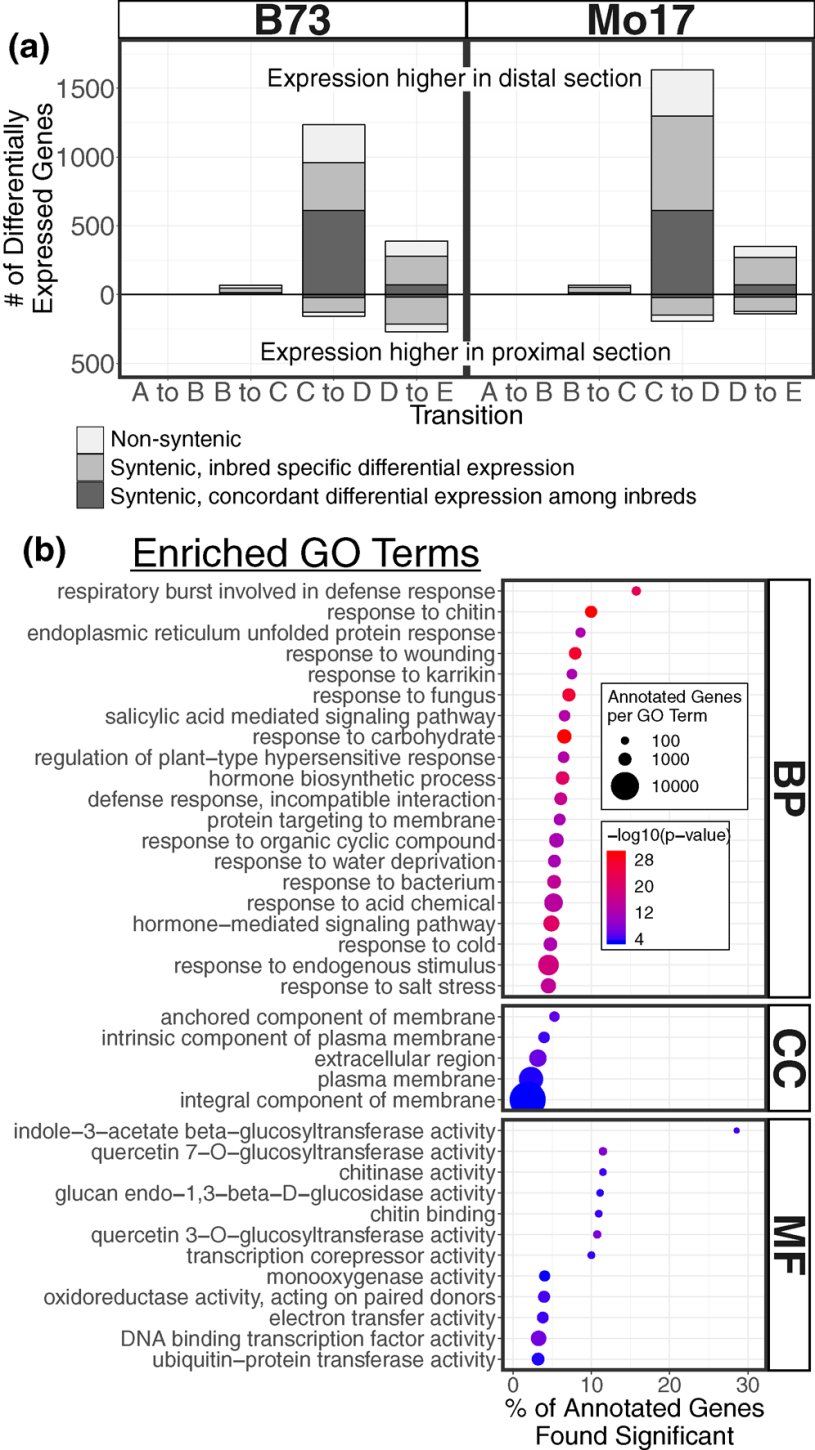
Differential gene expression along the lengths of maize silks. (a) Numbers of differentially expressed genes (DEGs) between neighboring silk sections are shown for both B73 and Mo17, with bar shades indicating syntenic expression states. (b) Significantly enriched Gene Ontology (GO) terms for syntenic DEGs between Sections C and D in both inbreds. Results are shown for separate enrichment tests that used the biological process (BP), cellular component (CC), and molecular function (MF) domains

To determine the extent to which gene expression varied along the silk length, differential expression analyses were performed for each of the four sets of neighboring silk sections (i.e., A vs. B, B vs. C, etc.). Within husk‐encased tissue, few genes were identified as being differentially expressed between adjacent sections (false discovery rate < 0.05 and log_2_ fold change > 1) (Figure 2a, Supplemental Table S2). In the A–B transition, only two B73 and three Mo17 DEGs were identified. Moreover, only 71 B73 and 68 Mo17 DEGs were identified for the B–C transition, highlighting high similarity among these three contiguous husk‐encased tissues.

In contrast, pronounced expression changes occurred in the transition between the most distal husk‐encased section (C) and the most proximal emerged section (D). Clear majorities of the 1,393 B73 (88.7%) and 1,828 Mo17 (89.4%) DEGs between these sections had greater expression in the emerged as opposed to husk‐encased silk tissue. Modest gene expression differences occurred between the two emerged sections (i.e., D vs. E), with 658 B73 and 491 Mo17 DEGs identified. However, considerable proportions (B73: 260 out of 658; Mo17: 291 out of 491) of these DEGs were also differentially expressed in the same way (i.e., up‐ or downregulated in the C–D and D–E transitions). In fact, expression along the silk length almost never changed direction across the series of transitions (Supplemental Figure S4). These trends were observed for both the syntenic and nonsyntenic gene sets (Figure 2a). However, nonsyntenic genes were differentially expressed in Sections C vs. D at a considerably lower rate than syntenic genes (*p* < 2.2 × 10^–16^ for both B73 and Mo17).

The growing year in which samples were collected had a substantial influence on gene expression (Figure 1c). To determine if these influences affected gene expression uniformly along the silk length, differential expression analyses were performed between samples from different growing years for each silk section. These revealed that gene expression was evenly affected along the silk length in B73, as similar numbers of genes were identified as differentially expressed between the two growing years in the inbred's five sections. Conversely, Mo17 had more genes identified as differentially expressed between growing years in its most distal sections (i.e., Sections D and E)(Supplemental Figure S5a). Additionally, Mo17 had fewer DEGs between Sections C and D in 2015 than in 2014 (Supplelmental Figure S5b). As such, growing year had a strong yet unequal influence on gene expression in the two inbreds. Specifically, Mo17 was more sensitive to environmental differences in its emerged as opposed to husk‐encased tissues. Nevertheless, the prevailing expression trends that were revealed when samples from different growing years were jointly analyzed (Figure 2a) still existed when they were analyzed individually (Supplemental Figure S5b). To maximize the statistical power and enrich the data for growing year‐agnostic trends, subsequent analyses were performed by jointly analyzing the eight bioreplicate samples collected across the two growing years.

Expression patterns along the silk length were similar for the B73 and Mo17 inbreds. Over 90% (91.4% in B73; 90.2% in Mo17) of expressed genes were not differentially expressed at any of the four silk section transitions. Of the genes displaying differential expression, consistent numbers were identified at each transition between inbreds (Figure 2a, Supplemental Figure S4). Moreover, a large proportion of DEGs were syntenic genes with differential expression in both inbreds. In particular, 632 of the 1,393 B73 (45.4%) and 1,828 Mo17 (34.6%) Section C–D DEGs were syntenic genes that changed expression concordantly in the two inbreds (i.e., up‐ or downregulated in both inbreds) (Figure 2a). Additionally, the fold change differences observed for syntenic genes in B73 and Mo17 at the Section C–D transition were highly correlated (*r* = 0.56; *p* < 2.2 × 10^–16^) (Supplemental Figure S6a). As such, syntenic genes that were differentially expressed in only one inbred often had expression changes in the same direction in the other inbred, though not exceeding the thresholds set for differential expression (i.e., false discovery rate < 0.05 and an absolute fold change > 2) (Supplemental Figure S6b).

To assess the biological relevance of genes for which expression differed along the spatiotemporal gradient, GO enrichment tests were conducted on the transitions across which expression was most dynamic and on the biological responses that were most conserved. As such, GO enrichment tests were performed on the 632 syntenic genes that were differentially expressed between husk‐encased Section C and emerged Section D concordantly for the two inbreds. The majority of GO terms significantly enriched in this analysis are related to biotic and abiotic stress responses (Figure 2b). An analogous evaluation of GO term enrichment was performed for nonsyntenic Section C vs. Section D DEGs of B73 and revealed the same biological trends (Supplemental Figure S7). Within the biological process GO domain, defense‐related GO terms that involve the recognition of pathogens or pests (e.g., response to fungus, chitin, wounding, and carbohydrate) or hormone‐mediated signaling (e.g., SA‐mediated signaling pathway) were highly enriched. Similarly, within the molecular function GO domains, terms related to pathogen or pest interaction (e.g., chitinase activity, chitin binding, glucan endo‐1‐3‐β‐*D*‐glucosidase activity) are enriched. An enrichment test of cellular component GO domains revealed that these DEGs are often located in the plasma membrane or extracellular region. To identify the subset of DEGs between silk sections C and D that were predicted to be localized within the plasma membrane or to be secreted from the cell, amino acid sequences were scanned for membrane signal sequences by SignalP5.0 (Almagro Armenteros et al., 2019) and transmembrane helices by TMHMM Version 2.0 (Krogh et al., 2001). In all, 287 (20.6%) and 322 (17.6%) B73 and Mo17 DEGs respectively had a signal sequence (Supplemental Figure S8a). This corresponds to a 2.16‐fold enrichment (*p* = 5.4 × 10^–37^) for B73 and 1.96‐fold enrichment (*p* = 7.5 × 10^–33^) for Mo17, given the total number of signal sequence‐containing genes in the genomes of the two inbreds revealed via a hypergeometric test. Of these signal sequence‐containing DEGs, 62 B73 and 87 Mo17 DEGs were predicted to be localized in the plasma membrane, as they also have at least one predicted transmembrane α‐helix domain. Therefore, 225 and 235 of these gene products are likely to be secreted from the cell in B73 and Mo17, respectively.

To gather insights regarding the potential role of these putatively secreted gene products, protein domain scans were made and identified a relative overabundance of CRPs, one of the best studied groups of secreted proteins. Cystine‐rich peptides are particularly abundant during plant reproduction and function as signaling peptides in fertilization, seed development, and the protection of reproductive tissue (Bircheneder & Dresselhaus, 2016; Marshall, Costa, & Gutierrez‐Marcos, 2011). Of the over 200 DEGs in each inbred that were putatively secreted from the cell, hidden Markov models (Silverstein et al., 2007) revealed that 34 and 33 encode CRPs in B73 and Mo17, respectively (Supplemental Figure S8a). These putative CRPs represent a diverse collection of genes that includes lipid transfer proteins and both chitinase and heveins, all of which have well‐established roles in plant defense (Jashni, Mehrabi, Collemare, Mesarich, & de Wit, 2015; Liu et al., 2015) (Supplemental Figure S8b). Moreover, several of these CRPs were present in only one genome (i.e., nonsyntenic) or either uniquely or differentially expressed between the inbreds (Supplemental Figure S8c), reflecting potential differences in defense responses.

### 3.3 Relationship between husk encasement status and stress response

**FIGURE 3:**
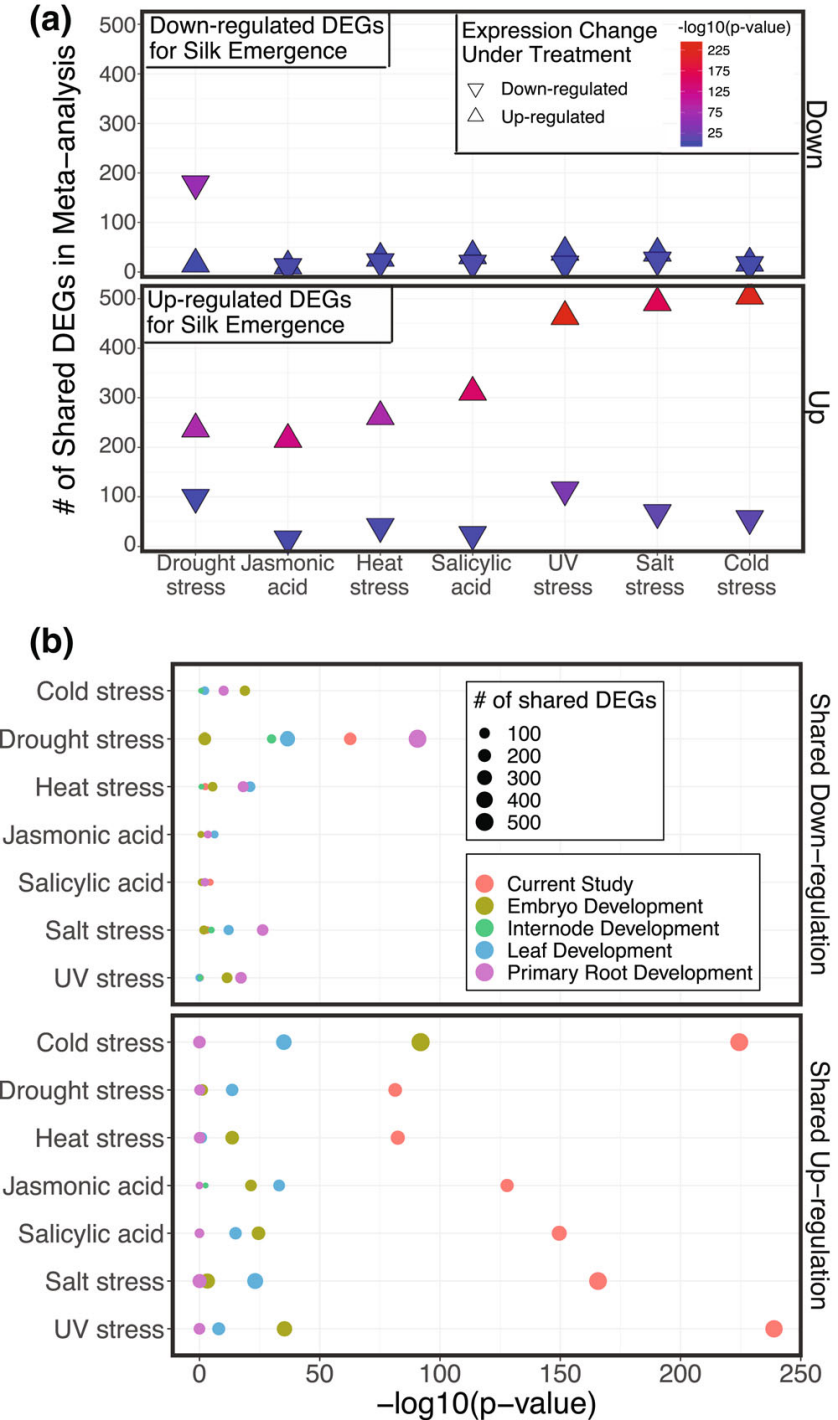
B73 differentially expressed genes (DEGs) between husk‐encased and emerged maize silks overlap with DEGs identified in other stress transcriptome studies. (a) Results of 28 meta‐analytic tests, where the outcomes for silks are compared with outcomes from seven published studies (Supplemental Table S3). The upper and lower panels divide tests based on the direction of expression change between encased and emerged silks; *n* = 1,076 and n = 2,230, respectively. Numbers of overlapping DEGs are conveyed by the position of the triangles, with colors corresponding to the −log_10_ *p*‐values in Fisher's exact tests. Triangle direction indicates the response to the treatment imposed within the published study. The total numbers of up‐ and downregulated DEGs identified in the published studies are shown in Supplemental Table S4. (b) Significance of overlap, according to Fisher's exact tests, with stress‐related DEGs for the DEG lists identified in the current study. For comparison, the same tests were used to determine the overlap between the stress‐related DEG lists and the DEG lists derived from developmental contrasts within several maize tissues, which are collectively meant to serve as negative controls

The abundance of DEGs enriched for stress‐related responses between Sections C and D suggests that emerged silks undergo dramatic gene expression changes in response to higher levels of environmental stress. This hypothesis was tested by determining whether DEGs between husk‐encased and emerged silks are also differentially expressed in maize seedlings under various abiotic and biotic stress conditions. Differentially expressed gene lists were gathered from previous transcriptome studies conducted with seedlings exposed to five abiotic (drought, heat, ultraviolet light, salt, and cold) and two biotic (JA and SA treatment) stress‐related conditions (Supplemental Table S3). Highly significant overlaps (*p* < 1 × 10^–75^) in DEG sets were observed between the 2,230 B73 DEGs upregulated in emerged silks and the genes identified as upregulated in seedlings under the seven unique stresses (Figure 3a, Supplemental Table S4). By comparison, there was a lack of overlap between the seedling stress‐responsive gene sets and the genes downregulated in response to silk emergence, with the exception of the drought‐downregulated gene list, with which those genes had significant and substantial overlap (Figure 3a, Supplemental Table S4).

Differentially expressed genes identified between emerged vs. encased silk sections could reflect differences in development rather than to exposure to environmental stresses. Therefore, as negative controls, the DEG lists generated from developmental contrasts (Supplemental Table S3) for each of four organs (embryos, internodes, leaves, and primary roots) were tested against the DEG lists generated from the seven seedling stresses (Figure 3b). For the list of genes upregulated by silk emergence, the *p*‐values were universally more than 70 orders of magnitude smaller across all comparisons between the current study and the negative controls, indicating that emergence‐induced gene expression in silks largely reflects a response to biotic and abiotic stresses (Figure 3b). These analyses were repeated for the encased vs. emerged Mo17 DEG set and recapitulated the B73 results precisely (Supplemental Figure S9), demonstrating that the response to exposure stress upon emergence is shared by these two diverse inbreds.

The observation that previously identified stress‐responsive genes largely overlap with genes responsive to silk emergence suggests that stress‐related TFs may be responsible for some of the expression changes observed between husk‐encased and emerged silks. Among all maize TF families, six were enriched for differential expression between husk‐encased and emerged silks (Supplemental Figure S10a), including the AP2‐EREBP, MYB, NAC, WRKY, ZIM, and HSF stress‐related TF families (Chen et al., 2012; Li, Ng, & Fan, 2015; Mizoi, Shinozaki, & Yamaguchi‐Shinozaki, 2012; Nakashima, Takasaki, Mizoi, Shinozaki, & Yamaguchi‐Shinozaki, 2012; Pauwels & Goossens, 2011; Scharf, Berberich, Ebersberger, & Nover, 2012). Aside from the MYB TF family, all of these families were predominately upregulated in emerged silks (Sections D and E), as well as in the published work cited in Supplemental Table S3 involving stressed seedlings (Supplemental Figure S10b).

### 3.4 Expression differences within the hormone signaling and metabolite biosynthesis pathways involved in defense

**FIGURE 4:**
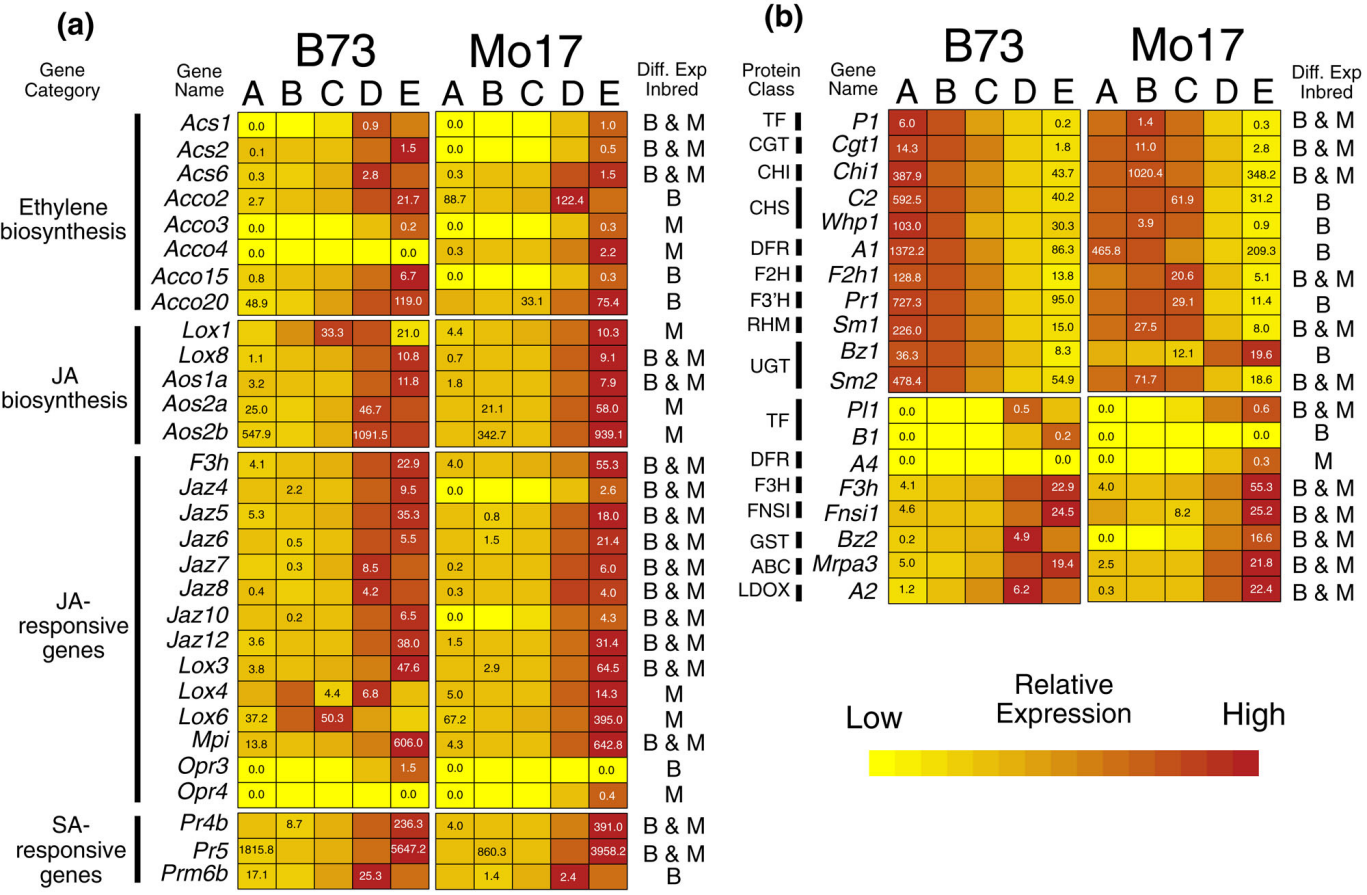
Spatial variability in defense hormone‐related and flavonoid gene expression along the maize silk length is largely conserved between B73 (B) and Mo17 (M). Heatmaps show the relative change in expression across the five silk sections within an inbred for (a) defense‐related genes and (b) flavonoid genes. Genes are grouped by pathways, with abbreviations and additional details provided in Supplemental Table S5. The transcript‐length‐normalized reads per million reads (RPKM) values of the tissues with the highest (white text) and lowest (black text) expression within an inbred for each gene are shown in both heatmaps. The inbreds identified as differentially expressed for each gene are also indicated. JA, jasmonic acid; SA, salicylic acid; TF, transcription factor; CGT, *C*‐glucosyltransferase; CHI, chalcone isomerase; CHS, chalcone synthase; DFR, dihydroflavonol reductase; F2H, flavanone 2‐hydrolxylase; F3’H, flavonoid 3’‐hydroxylase; RHM, rhamnose synthase; UGT, UPD‐glycosyltransfersae; GST, glutathione *S*‐transferase; ABC, ATP binding cassette transporter; LDOX, leucoanthocyanidin dioxygenase

Biotic stress responses observed during silk emergence were further analyzed by identifying DEGs between husk‐encased and emerged silks that are associated with JA, ethylene, and SA, three key hormones involved in the regulation of defense signaling (Li, Han, Feng, Yuan, & Huang, 2019). Numerous genes involved in the biosynthesis of ethylene and JA were upregulated in B73 and Mo17 emerged silks (Figure 4a, Supplemental Table S5). These included the genes that encode ethylene‐related enzymes 1‐aminocyclopropane‐1‐carboxylate synthase and 1‐aminocyclopropane‐1‐carboxylate oxidase, and the 13‐ and 9/13‐lipoxygenase (LOX) and allene oxide synthase JA‐related enzymes. No SA biosynthesis genes were identified as being differentially expressed between husk‐encased and emerged silks. However, the SA‐induced pathogenesis‐related genes *Pr4b*, *Pr5*, and *Prm6b* (Ziemann et al., 2018) had significantly higher expression in emerged silks. Many JA wound‐inducible genes (Borrego & Kolomiets, 2016) were also upregulated, including several 9‐LOXs and Type I 12‐ oxo‐11,15‐phytodienoic acid reductases presumed to be involved in the production of 10‐oxo‐11‐phytoenoic acid and 10‐oxo‐11,15‐phytodienoic acid. Downstream indications of ethylene‐mediated signaling were observed, including the upregulation of ethylene‐responsive AP2/EREBP TFs (Müller & Munné‐Bosch, 2015) (Figure 4a, Supplemental Table S5).

A large set of biosynthesis genes involved in the production of defense‐related specialized metabolites was identified as being differentially expressed between husk‐encased and emerged silks (Supplemental Table S6), including genes involved in the production of benzoxazinoid (Bx), oxylipin, terpenoid, and maysin metabolites. Of the 14 Bx genes (Zhou, Richter, & Jander, 2018) that participate in benzoxazinoid production, four genes involved in the earlier stages of the pathway were downregulated (*Bx2*, *Bx3*, *Bx4*, and *Bx5*), whereas four genes involved in the later stages were upregulated (*Bx10*, *Bx11*, *Bx13*, and *Bx14*) in emerged silks. Many of the known biosynthetic genes involved in the production of “death acids”, or 9‐LOX‐derived cyclopente(a)nones, with direct phytoalexin activity (Christensen et al., 2015), were upregulated in emerged silks. Notably, *Lox3*, which is required for host resistance to *Aspergillus flavus* (Gao et al., 2009), was induced (Figure 4a). Among the terpenoid biosynthesis genes, three sesquiterpene synthase (*Tps8*, *Tps9*, and *Tps10*) and two ent‐kaurene synthase (*Ks2* and *Ks4*) genes were upregulated in emerged silks (Supplemental Table S6).

*Pericarp color1*, an R2R3‐MYB TF (Grotewold, Drummond, Bowen, & Peterson, 1994) that transcriptionally regulates structural genes involved in the production of maysin and other 3‐deoxyflavonoid pathway genes, decreased in expression along the silk length (Figure 4b, Supplemental Table S5). In agreement with its key regulatory role, the changes observed for *Pericarp color1* were accompanied by a decrease in the expression of maysin biosynthesis genes (Figure 4b, Supplemental Figure S11). In fact, all genes in the pathway were differentially expressed between husk‐encased and emerged silks in at least one of the two inbreds (Supplemental Figure S11). Moreover, the only maysin biosynthesis genes that were not differentially expressed in both inbreds were the flavonoid 3′‐hydroxylase *Red aleurone1* and the two chalcone synthase genes, *Colorless2* and *White pollen1*.

In addition to maysin biosynthesis genes, numerous other flavonoid pathway genes were differentially expressed along the silk length (Figure 4b, Supplemental Figure S11, Supplemental Table S5). This was presumably the result of expression changes to two additional key flavonoid regulatory genes, *Purple plant1*, which is an R2R3‐MYB TF, and *B1* (*Booster1*), a basic helix‐loop‐helix TF. These two proteins interact to form a complex that controls the expression of various 3‐hydroxyflavonoid (anthocyanin) pathway genes (Goff, Cone, & Chandler, 1992). *Purple plant1*and *Booster1*increase distally along the silk length, which generally follows the same trend observed among anthocyanin‐related biosynthetic genes (Figure 4b).

### 3.5 Differential expression between B73 and Mo17 follows spatiotemporal patterns

Up to this point, analyses of DEGs have been conducted in parallel for B73 and Mo17, with the reported results displaying quite similar overall trends for the two inbreds (Figure 2, Figure 4, Supplemental Figure S2, Supplemental Figure S4, Supplemental Figure S5, Supplemental Figure S6, and Supplemental Figure S11). Although it is certainly the case that B73 and Mo17 silks respond to developmental, physiological, and environmental cues with similar global responses, gene expression differentiating these two well‐diverged founder inbreds is prevalent. Indeed, in the principal component analysis (Figure 1), Principal Component 1 completely separated the 40 B73 and 40 Mo17 samples of this experiment and accounted for 71% of the variance in global gene expression explained by Principal Components 1 to 3. Thus the simultaneous high degrees of similarity and dissimilarity between the inbreds are seemingly paradoxical.

**FIGURE 5:**
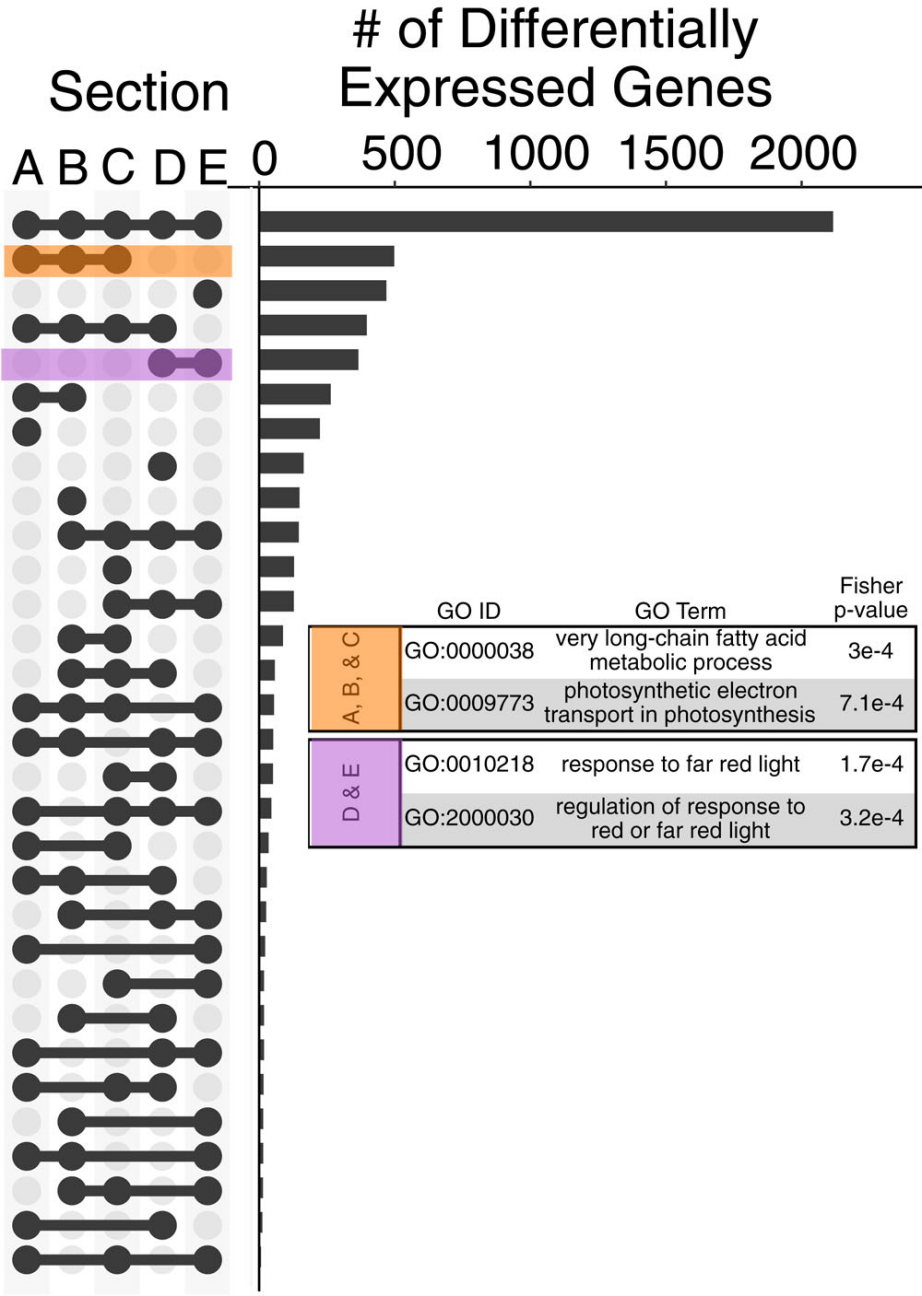
UpSet plot showing maize silk section trends for B73 vs. Mo17 differential gene expression. Darkened dots denote differential expression between the inbreds. When they are true for more than one section, they are connected by lines. Outcomes for 5,643 of the 24,928 syntenic gene pairs are reported on the basis of their differential expression in at least one silk section. Differential expression results for all 26 shared and all five single‐section patterns are displayed. The inset displays all biological process domain Gene Ontology (GO) terms found significantly enriched (*p* < 0.001) among differentially expressed genes in all encased (orange) or all emerged (purple) sections. Additional enrichment results are reported in Supplemental Table S7

To quantify the degree of expression differentiation between B73 and Mo17 and to consider spatiotemporal distribution patterns, DEGs between the inbreds were identified for each silk section via a conservative consensus approach that used both reference genomes (see Materials and Methods). Across 24,928 syntenic gene pairs, differential expression for B73 vs. Mo17 in at least one silk section was identified for 5,643 gene pairs. The intersections of DEG lists across silk sections A to E were then visualized to reveal the spatiotemporal patterns of differential expression (Figure 5). Interestingly, the most common pattern (37.5%) reflects shared differences across all five sections, with two of the other most common patterns being differential expression between the inbreds that was shared among all encased or between both emerged silk sections. More generally, contiguity across silk sections is a broad trend: among the 15 patterns with no discontiguities, 14 occupy the top 14 ranks (92.1% of DEGs collectively), with only the contiguously shared C–D pattern (0.9%) mixed among the 16 discontiguous patterns (7.0% collectively) (see Supplemental Table S7 for pattern categories). As a statistical matter, multiple independent identifications assured the veracity of the observed DEG lists to an impeccable degree. As to their biological importance, these results suggest that hundreds of syntenic pairs have their expression differences controlled by *cis‐* and/or *trans‐*acting factors that respond to spatiotemporal cues, whether they be developmental, physiological, or environmental. This suggestion is further supported by the functionally relevant biological process GO term enrichments observed for a subset of patterns (Figure 5, Supplemental Table S7). For example, the shared DEGs across the three encased silk sections were enriched for the GO term, “very long chain fatty acid metabolic process”, which is biologically supported by known differences in cuticular lipid accumulation on encased silks of B73 and Mo17 (Loneman et al., 2017; Perera et al., 2010).

The observations that B73 vs. Mo17 differences across silk sections appear to be closely coordinated and functionally related explains, to some degree, the aforementioned similarity–dissimilarity paradox. Indeed, pairs of syntenic genes can be responsive to the same cues yet expressed differently between the two inbreds. In support of this idea, we observed that among the 632 DEGs distinguishing silk Sections C and D concordantly in B73 and Mo17 (Figure 2a), 371 (58.7%) and 117 (18.5%) were differentially expressed between inbreds in at least one or in all five silk sections respectively (Figure 5). An additional explanation for the paradox has already been invoked: syntenic genes that are deemed to be differentially expressed in only one inbred typically show the same trend in the other (Figure 2, Supplemental Figure S6).

### 3.6 Syntenic gene coexpression network analysis

**FIGURE 6:**
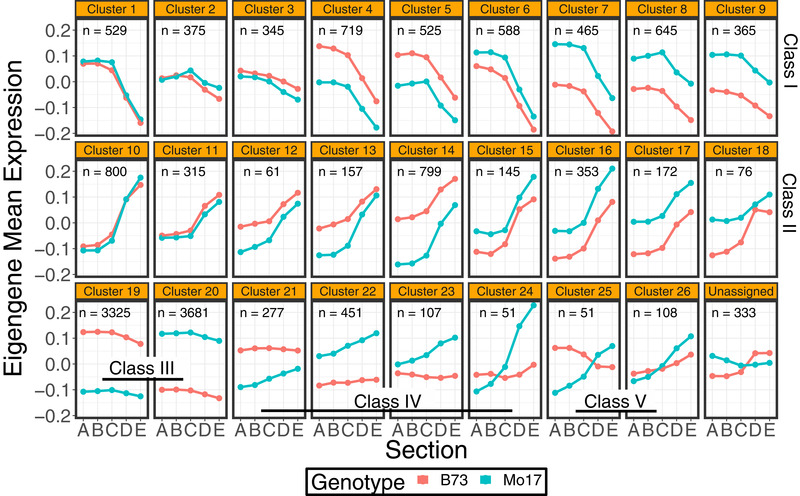
Patterns of coexpression clusters identified via network analysis of syntenic genes expressed in both B73 and Mo17 maize inbreds (*n* = 15,818). Average eigengene expression trends for both inbreds are shown for 26 co‐expression clusters, which are further categorized into five classes

To further identify and distinguish between shared and distinct gene expression patterns in the two inbreds, we constructed a gene coexpression network (GCN) with the log_2_‐normalized expression values of expressed syntenic genes from all 80 samples collected in this investigation. This approach took full advantage of the spatiotemporal transcriptome profiles and detected gene sets with correlated expression across all five silk sections and between the two inbreds. The resulting GCN consisted of 15,485 genes partitioned into 26 gene clusters (Figure 6, Supplemental Table S8). Collectively, the gene clusters were assigned to five classes according to the attributes of their average eigengene expression patterns across inbreds and silk sections: Class I: decreasing in both inbreds (Clusters 1–9); Class II: increasing in both inbreds (Clusters 10–18); Class III: constant but higher in one of the inbreds (Clusters 19–20); Class IV: constant in B73 and increasing in Mo17 (Clusters 21–24); or Class V: changing in opposite directions or at different rates in the two inbreds (Clusters 25 and 26).

Class I to III clusters encompass a large majority of the genes in the GCN (14,440 out of 15,485) and, notably, appear to have shared spatiotemporal patterns in both inbreds, even though their expression intensities may vary (Figure 6). These observations reiterate similar trends revealed through differential expression analyses of the two inbreds: differential expression between inbreds often occurs across multiple, if not all, silk sections and exists for genes exhibiting spatiotemporally dynamic expression in both inbreds (Figure 5). Together, the expression patterns of B73 and Mo17 genes in Class I to III clusters congruently increased or decreased along the silk length, probably in response to silk emergence or some additional spatiotemporal gradient.

To determine which Class I to III clusters showed the greatest expression changes along the silk length, DEGs between husk‐encased and emerged silks were counted within each of the clusters (Supplemental Figure S12). Of 563 genes that were downregulated in emerged relative to encased tissues, 322 (57.2%) belong to Clusters 4 and 6. Moreover, 1,184 (69.6%) of the 1,701 upregulated genes in the GCN are part of Clusters 10, 14, and 16. These three upregulated clusters are all highly enriched for stress‐related biological processes (Table 1, Supplemental Table S9), including many of the GO terms identified among the syntenic DEGs in both B73 and Mo17 at the Section C–D transition (Figure 2b). Moreover, 591 of the 956 (61.8%) syntenic genes that were upregulated in both emerged silks and at least one of the seven seedling stress conditions (Figure 3a) belong to the same three upregulated clusters. As such, Clusters 10, 14, and 16 encompass many of the stress response‐related genes upregulated during silk emergence.

The two downregulated clusters (4 and 6) are not enriched for stress‐related GO terms (Table 1, and Supplemental Table S9). Instead, GO terms related to cellular development were enriched. For Cluster 4, the enriched GO terms include regulation of cell size and auxin transport, which suggests that at least some of the Cluster 4 genes are involved in cell elongation. Indeed, Cluster 4 includes the auxin efflux carriers *Brachytic2*, *Pin1* (*PIN‐FORMED1*), *Pin2* (*PIN‐FORMED2*), and *Pin10* (*PIN‐FORMED10*), as well as the cell‐wall loosening protein *Alpha expansin4* and two cellulose synthases, *Csa6* and *Csa9*. The enriched GO terms for Cluster 6 (e.g., microtubule‐based movement and cytoskeleton organization) highlight their potential involvement in cell division, which is consistent with the presence of genes encoding the cohesion complex protein (*Absence of first division1*), the cyclin dependent kinase (*Cell division control2*), the α‐tubulin protein (*Tua3*), and the β‐tubulin proteins (*Tub5* and *Tub6*). Collectively, Clusters 4 and 6 support the developmental gradient observed along the silk length at 3 d after silk emergence, in which cell division and elongation ceased in emerged silks (Fuad‐Hassan et al., 2008).

**FIGURE 7:**
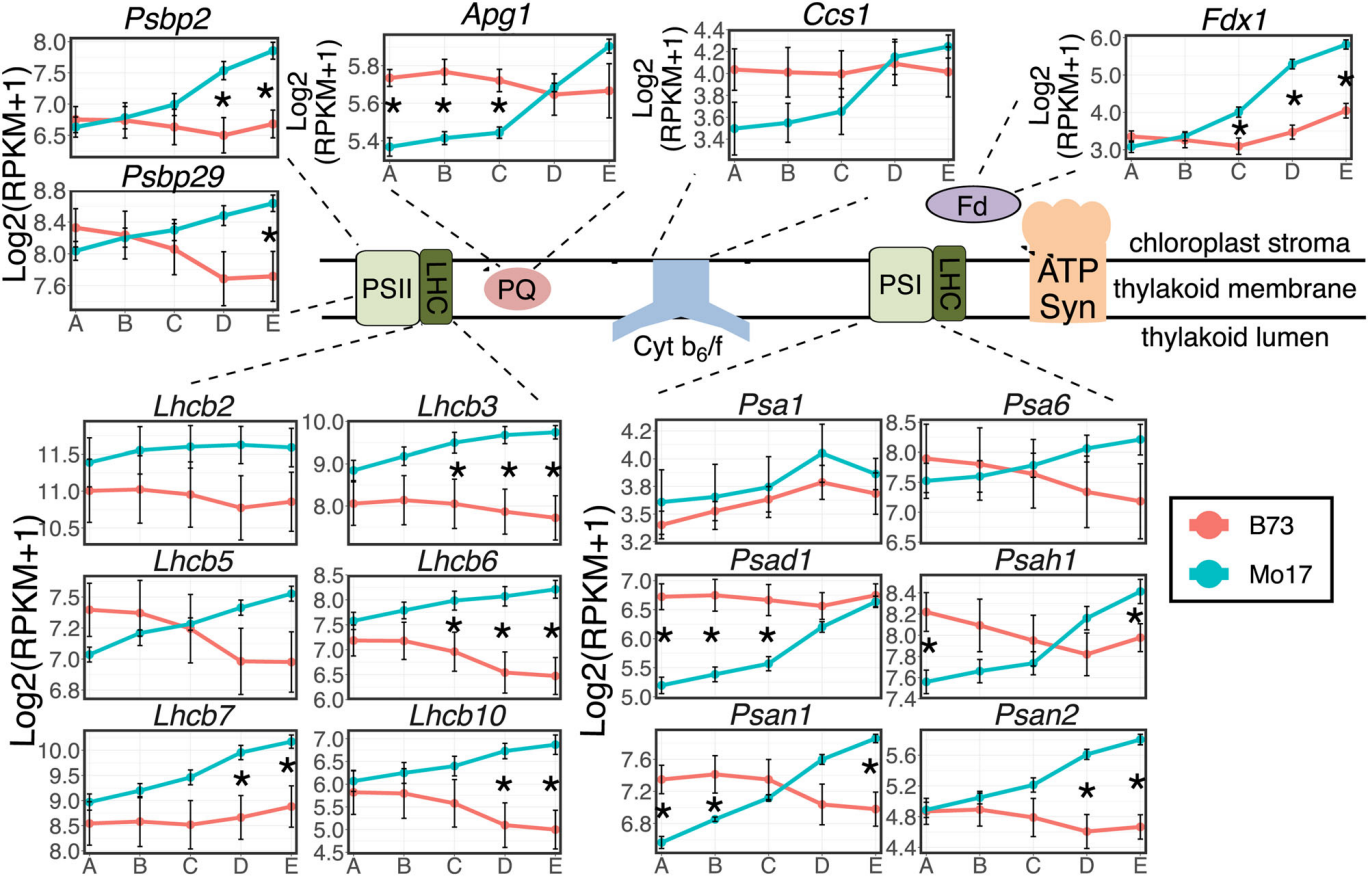
Expression pattern differences for genes encoding proteins of the light‐dependent reactions of photosynthesis in B73 and Mo17 maize inbreds. Asterisks denote statistical differences (*p* < 0.05) between the two inbreds for a particular silk section, as computed by a two‐tailed Student's *t*‐test. PSII, Photosystem II; LHC, light‐harvesting complex; PQ, plastoquinone; Cyt b_6_/f, cytochrome b_6_f complex; PSI, Photosystem I; Fd, ferredoxin; ATP Syn, adenosine 5′‐triphosphate synthase; RPKM, transcript‐length‐normalized reads per million reads

Class IV and V clusters represent gene sets with distinct expression patterns along the silk length between the two inbreds (Figure 6). Gene Ontology enrichment tests of these clusters revealed a similar set of enriched biological process terms among Clusters 23 to 26; specifically photosynthesis‐ and light response‐related GO terms (Table 1). These enrichments indicate that photosynthesis‐related genes within these clusters remained mostly constant or decreased along the silk length in B73, whereas in Mo17, they exhibited an increase in expression in emerged silks (Figure 7). Cluster 23 is particularly rich in light‐harvesting complex genes that encode six chlorophyll a/b binding proteins, three Photosystem I subunits, and two Photosystem II subunits (Figure 7). Similar GO terms enriched in Clusters 23 to 26 are also enriched among the 498 and 366 DEGs between the two inbreds specifically in the three husk‐encased or two emerged sections, respectively (Figure 5).

**TABLE 1:** Highly enriched Gene Ontology terms for 11 particularly interesting gene coexpression network clusters

Cluster	GO ID	GO Term	*p*‐value
Cluster 4^a^	GO:0042256	Mature ribosome assembly	1.2 × 10^–12^
	GO:0009825	Multidimensional cell growth	1.6 × 10^–10^
	GO:0043481	Anthocyanin accumulation in response to ultraviolet light	3.5 × 10^–9^
	GO:0008361	Regulation of cell size	4.9 × 10^–8^
	GO:0045229	External encapsulating structure organization	5.9 × 10^–8^
Cluster 6^a^	GO:0007018	Microtubule‐based movement	3.1 × 10^–7^
	GO:0007010	Cytoskeleton organization	2.4 × 10^–5^
	GO:0007020	Microtubule nucleation	1.9 × 10^–4^
Cluster 10^a^	GO:0010200	Response to chitin	1.0 × 10^–30^
	GO:0009620	Response to fungus	1.3 × 10^–21^
	GO:0009743	Response to carbohydrate	7.5 × 10^–21^
	GO:0009755	Hormone‐mediated signaling pathway	1.5 × 10^–15^
	GO:0042446	Hormone biosynthetic process	3.4 × 10^–15^
Cluster 14^a^	GO:0010200	Response to chitin	4.9 × 10^–11^
	GO:0009743	Response to carbohydrate	1.0 × 10^–9^
	GO:0009744	Response to sucrose	5.3 × 10^–8^
	GO:0046148	Pigment biosynthetic process	1.1 × 10^–7^
	GO:0042446	Hormone biosynthetic process	2.1 × 10^–7^
Cluster 16^a^	GO:0071215	Cellular response to abscisic acid stimulus	4.6 × 10^–6^
	GO:0009626	Plant‐type hypersensitive response	3.3 × 10^–5^
	GO:0045088	Regulation of innate immune response	3.9 × 10^–5^
	GO:0009863	Salicylic acid‐mediated signaling pathway	5.2 × 10^–5^
	GO:0030968	Endoplasmic reticulum unfolded protein response	8.9 × 10^–5^
Cluster 21^b^	GO:0009083	Branched‐chain amino acid catabolic process	8.2 × 10^–4^
Cluster 22^b^	GO:0018130	Heterocycle biosynthetic process	6.3 × 10^–5^
	GO:0070940	Dephosphorylation of RNA Polymerase II *C*‐terminal domain	6.6 × 10^–5^
Cluster 23^b^	GO:0009768	Photosynthesis, light harvesting in Photosystem I	1.0 × 10^–30^
	GO:0018298	Protein–chromophore linkage	1.5 × 10^–25^
	GO:0019344	Cysteine biosynthetic process	3.7 × 10^–18^
	GO:0009637	Response to blue light	1.5 × 10^–16^
	GO:0010218	Response to far red light	1.3 × 10^–14^
Cluster 24^b^	GO:0009773	Photosynthetic electron transport in Photosystem I	8.6 × 10^–8^
	GO:0019288	Isopentenyl diphosphate biosynthetic process	1.5 × 10^–6^
	GO:0006354	DNA‐templated transcription, elongation	1.0 × 10^–4^
	GO:0007286	Spermatid development	2.2 × 10^–4^
	GO:0015995	Chlorophyll biosynthetic process	3.6 × 10^–4^
Cluster 25^b^	GO:0019684	Photosynthesis, light reaction	2.6 × 10^–8^
	GO:0006754	ATP^c^ biosynthetic process	9.0 × 10^–7^
	GO:0019344	Cysteine biosynthetic process	1.6 × 10^–5^
	GO:0009657	Plastid organization	8.6 × 10^–5^
	GO:0019673	GDP^c^‐mannose metabolic process	3.2 × 10^–4^
Cluster 26^b^	GO:0042793	Plastid transcription	3.8 × 10^–9^
	GO:0009902	Chloroplast relocation	4.1 × 10^–7^
	GO:0010027	Thylakoid membrane organization	6.1 × 10^–7^
	GO:0019682	Glyceraldehyde‐3‐phosphate metabolic process	1.1 × 10^–4^
	GO:0006739	NADP^c^ metabolic process	1.5 × 10^–4^

^a^ These GCN clusters were enriched for encased vs. emerged DEGs.

^b^ These GCN clusters were enriched for genotypically divergent patterns of differential expression. ATP, adenosine 5′triphosphate; GDP, guanosine diphosphate; NADP, nicotinamide adenine dinucleotide phosphate.

## 4 DISCUSSION

### 4.1 Silk emergence as a model for molecular investigations of stress response

Silk transcriptomic responses during emergence were characterized precisely, enabled by the long lengths of silks and the ease with which emerged and husk‐encased tissue can be differentiated. The abruptness of the transition from unexposed to exposed offers a unique opportunity to study the dynamics related to the preparedness for and the response to environmental stress. For example, differential reactions between encased and emerged silks to abiotic and biotic stresses could be tested in relation to controlled environment manipulations (e.g. drought, light, or temperature stress), pest infestation, or pathogen inoculation. Indeed, numerous stress response signatures were identified among DEGs between husk‐encased and emerged tissue, including the activation of defense hormone signaling, cellular secretion pathways, and defense metabolite biosynthesis. Moreover, the number and variety of stresses mimicked by silk emergence in the meta‐analysis of seedling stress treatments was particularly striking (Figure 3, Supplemental Table S3). These observations, along with the enrichment for differential expression of numerous stress‐related TF families, indicates that diverse stress pathways were activated (Supplemental Figure S10).

These observations may have been expected because silks, like most stigmatic organs in other plant species, have proportionally large surface areas and are well‐nourished (Heslop‐Harrison, 1992). These qualities are critical for supporting pollen germination and pollen tube growth toward the ovule, but also make them a target for both pests and pathogens. Silks face additional challenges because of their long lengths, which can be upwards of 20 cm (Dresselhaus, Lausser, & Márton, 2011), and high water content (∼90%). When silks emerge from husk leaves for pollen reception, multiple stresses are experienced at sharply increased intensities, which probably accounts for the strong overlaps between emerged vs. husk‐encased DEGs in silks and DEGs responsive to abiotic and biotic seedling stress (Figure 3b, Supplemental Figure S9). For example, desiccation prevention following emergence, even under drought conditions, is important because it helps maintain pollen receptivity if pollination is delayed (Bassetti & Westgate, 1993). Moreover, protection from pathogens and pests (e.g., via JA‐mediated stress responses) is crucial because many species target silks as a primary food source and/or use their resources and structure for passage to the developing kernels (Ortega Corona, 1987; Pechanova & Pechan, 2015).

Further relating to pathogen and pest interactions, numerous genes involved in hormone‐mediated defense signaling and defense metabolite biosynthesis were differentially expressed during silk emergence (Figure 6). In accordance with an expectation of increased gene expression under increased threat, these genes were typically upregulated in emerged silks and belonged to GCN Clusters 10, 14, and 16, which share increasing expression along the silk length in both inbreds (Figure 5, Supplemental Table S8). In contrast, all structural and regulatory genes involved in maysin biosynthesis, an intensely studied and agronomically important antibiosis compound, were downregulated during silk emergence and belonged to clusters with decreasing expression along the silk length (Figure 4). With the exception of *Chalcone isomerase1*, which belongs to Cluster 6, all structural genes in the maysin biosynthesis pathway belong to Cluster 4 (Figure 5, Supplemental Table S8). Cluster 4 also contains *Pericarp color1*, the R2R3‐MYB TF known to directly control maysin biosynthesis, which has previously been shown to directly interact with the promoters of five of the six maysin structural genes in Cluster 4, including *Colorless2*, *F2H1*, *Red aleurone1*, *Salmon silk1*, and *Salmon silk2* (Casas et al., 2016; Morohashi et al., 2012). Interestingly, Clusters 4 and 6 are both enriched for GO terms associated with development (Supplemental Table S9). As such, maysin gene expression may be developmentally regulated, potentially explaining the downregulation observed in more distal silk tissues. If transcript abundances of these genes truly reflect their metabolic activities, maysin would be produced at higher rates in husk‐encased tissues, providing the silk with protective maysin metabolites prior to the elongation and subsequent emergence of that tissue into the external environment. Numerous studies have examined maysin concentrations in silks (Byrne et al., 1996; Casas et al., 2016; Szalma et al., 2005). However, because these experiments were conducted with entire silks, no spatial conclusions can be drawn. As such, inclusion of spatiotemporal dissection in future biochemical investigations may provide useful insights into the production of this important compound. Indeed, intriguing spatiotemporal patterns of maysin expression were recently uncovered via mass spectrometry imaging of cross‐sections of maize leaves, showing preferential accumulation of maysin in the adaxial epidermal layer only (Korte, Yandeau‐Nelson, Nikolau, & Lee, 2015).

### 4.2 Comparison of silk and leaf spatiotemporal expression patterns

According to the dynamic expression patterns of developmental genes, maize silks seem to possess a developmental gradient along their lengths similar to that of monocot leaves, wherein cell division and elongation are limited to basal regions (Sharman, 1942; Tardieu, Reymond, Hamard, Granier, & Muller, 2000). Kinematic analyses of cell division and elongation (Fuad‐Hassan et al., 2008) in silks have identified this same developmental gradient for cellular elongation at the stage that silk tissues were evaluated in this study (i.e., 3 d after silk emergence). As such, comparisons of the spatiotemporal expression patterns of silks and leaves may provide further insights into the unique and shared properties of both organs. Fortunately, these comparisons can be made, as expression patterns along the maize leaf have been examined across 10 fully contiguous sections (Pick et al., 2011) and within four nonadjacent sections (Li et al., 2010). Analogous to the silk, both increasing and decreasing expression patterns could be identified along the leaf length. Moreover, genes with their highest expression at the base were determined to be involved in development. Unlike in the silk, genes that increase in expression towards the tip are not generally involved in stress response but in photosynthesis instead. This was the case, despite leaves having to emerge from the whorl into the external environment in a process similar to silk emergence from husks. Genes with expression differences in the leaf across this transition may be involved in converting from a sink to a source tissue, rather than as a response to stress, although these possibilities are not mutually exclusive. Additionally, these same genes that increased in expression following emergence from the whorl returned to lower expression levels near the leaf tip. In contrast, genes that changed expression during silk emergence almost never returned to the levels observed in the husk‐encased portions of the silk (Supplemental Figure S4). Taken together, commonalities between silk and leaf gene expression in maize are likely to reflect similar developmental gradients along their lengths, even though their respective environmental gradients differ. This is probably a result of the leaf's substantial investment into photosynthesis‐related processes and the unique environmental contrasts silks experience as a result of their encasement and subsequent emergence from husks.

Importantly, previous research has shown that silk development is only analogous to the monocot leaf following emergence from husks. Cell division and elongation initially occur along the silk length, but after flowering, cell division progressively decreases from the silk tip to base and cell elongation only continues inside the husk encasement (Fuad‐Hassan et al., 2008). As such, we expect that transcriptomic examination of earlier developmental stages of silks would better reflect these patterns. Previous investigations have analyzed silk gene expression at more immature stages than the current study (Xu et al., 2012, 2013) but were focused on genes uniquely expressed in silks or that changed in expression during pollination, as opposed to along the silk length.

Potential targets for future investigations into silk development include genes in Clusters 4 and 6 of the syntenic GCN. For example, the auxin efflux carriers *Brachytic2*, *Pin1*, *Pin2*, and *Pin10*, which all resided in Cluster 4 and decreased in expression along the silk length, may control cell elongation and differentiation. These genes have been shown to have a strong effect on these two cellular processes in other maize tissues through the regulation of polar auxin transport (Carraro, Forestan, Canova, Traas, & Varotto, 2006; Multani et al., 2003). The application of our approach at earlier stages in development will be valuable in determining if these and other genes control development before silk emergence.

### 4.3 Biological relevance of variations in expression between B73 and Mo17

Variations in gene expression often underlie phenotypic differences observed among individuals of the same species (Chen et al., 2005; Cookson, Liang, Abecasis, Moffatt, & Lathrop, 2009). Thus the substantial genetic variation for gene expression in this study (Figure 1, Figure 5) is expected to predict ways in which B73 and Mo17 silks are phenotypically distinct. Although some are known (e.g., cuticular lipid variation), the majority are likely to be unknown, as inferred from the gap between the numerous biological processes showing functional enrichment (Supplemental Table S7) and the relative dearth of published studies on the biological attributes of silks. Moreover, these analyses did not examine nonsyntenic differences between the inbreds (Figure 2). In certain cases, such as for the CRPs, nonsyntenic variation could well be functionally important (Supplemental Figure S8).

An equally interesting example of gene sets with distinct expression patterns between inbreds was found in GCN Clusters 21 to 26, where increasing expression along the silk length existed mainly for Mo17 (Figure 6). Numerous genes from these clusters contribute to the biogenesis of the photosynthetic apparatus, which is counterintuitive, because most reproductive tissues are regarded as C sinks (White, Rogers, Rees, & Osborne, 2016). However, recent investigations have shown that reproductive tissues are capable of assimilating C, albeit at lower rates than C source tissues (Brazel & Ó’Maoiléidigh, 2019). Indeed, in our study of silks, most of the genes encoding proteins involved in the light‐dependent reactions of photosynthesis showed high expression. This accounts for measurable levels of chlorophyll being observed in silks (Žilić, Janković, Basić, Vančetović, & Maksimović, 2016) and indicates that oxygenic photosynthesis occurs in this tissue. Interestingly, expression differences between the inbreds for these genes suggest that Mo17 silks may rely on photosynthesis more than B73 silks, but the consequence of this difference is unclear. Although the photosynthetic apparatus creates high‐energy products used in the Calvin cycle, it can also confer photoprotection through nonphotochemical quenching (Kong, Xie, Sun, Si, & Hu, 2016). Both of these roles are expected to be beneficial to silks as they grow and emerge from husk encasement.

## 5 CONCLUSIONS

Spatiotemporal gene expression analysis along the lengths of maize silks has broadened our understanding of development and stress response in this agronomically important tissue. Numerous genes associated with mediating both biotic and abiotic stress responses were upregulated in emerged silks, including genes associated with the biosynthesis of protective anthocyanins, phytoalexins, benzoxazinoids, and stress hormones. Taken together with the fact that maize silks experience two very distinct environments (husk encasement vs. emergence), silks are an ideal organ in which to study stress responses. We envision this comprehensive tissue‐specific mini atlas in two maize inbred lines to be complementary to large‐scale gene expression atlases and permit functional hypothesis testing to study the biology within an organ. Moreover, this resource may be particularly valuable when paired with metabolomic datasets, especially when used in genetical genomic frameworks to dissect the genetic underpinnings of the phenotypic variation that distinguishes B73 and Mo17 silks.

## AUTHOR CONTRIBUTIONS

MYN and NL developed the original concept. KC, TD, ML, MYN, and NL developed the methodology. CM, KC, TD, ML, MYN, and NL conducted the investigation. CM, MYN, and NL carried out the formal analysis. NL provided the resources. Preparation of the original draft was done by CM, MYN, and NL. CM, KC, TD, ML, MYN, and NL reviewed and edited the final draft. MYN and NL contributed equally to this paper.

## CONFLICT OF INTEREST

The authors declare that there is no conflict of interest.

## Supporting information

Supporting Material

Supporting Material

Supporting Material

Supporting Material

Supporting Material

Supporting Material

Supporting Material

Supporting Material

Supporting Material

Supporting Material

Supporting Material

Supporting Material

Supporting Material

Supporting Material

Supporting Material

Supporting Material

Supporting Material

Supporting Material

Supporting Material

Supporting Material

Supporting Material

## Data Availability

Sequence data accompanied by detailed biosample metadata have been deposited in the NCBI Sequence Read Archive (PRJNA545496). All scripts used in this project are available on GitHub (https://github.com/cmcninch/maize_silk_spatio_temporal_gene_expression, accessed 16 July 2020).
