# Supplementary material for: A multigenotype maize silk expression atlas reveals how exposure‐related stresses are mitigated following emergence from husk leaves
Source: Plant Genome. 2020 Oct 14;13(3):e20040. doi: 10.1002/tpg2.20040 (PMC12806928; doi:10.1002/tpg2.20040)
Supplement: Supplementary file 1 [file TPG2-13-e20040-s001.pdf]

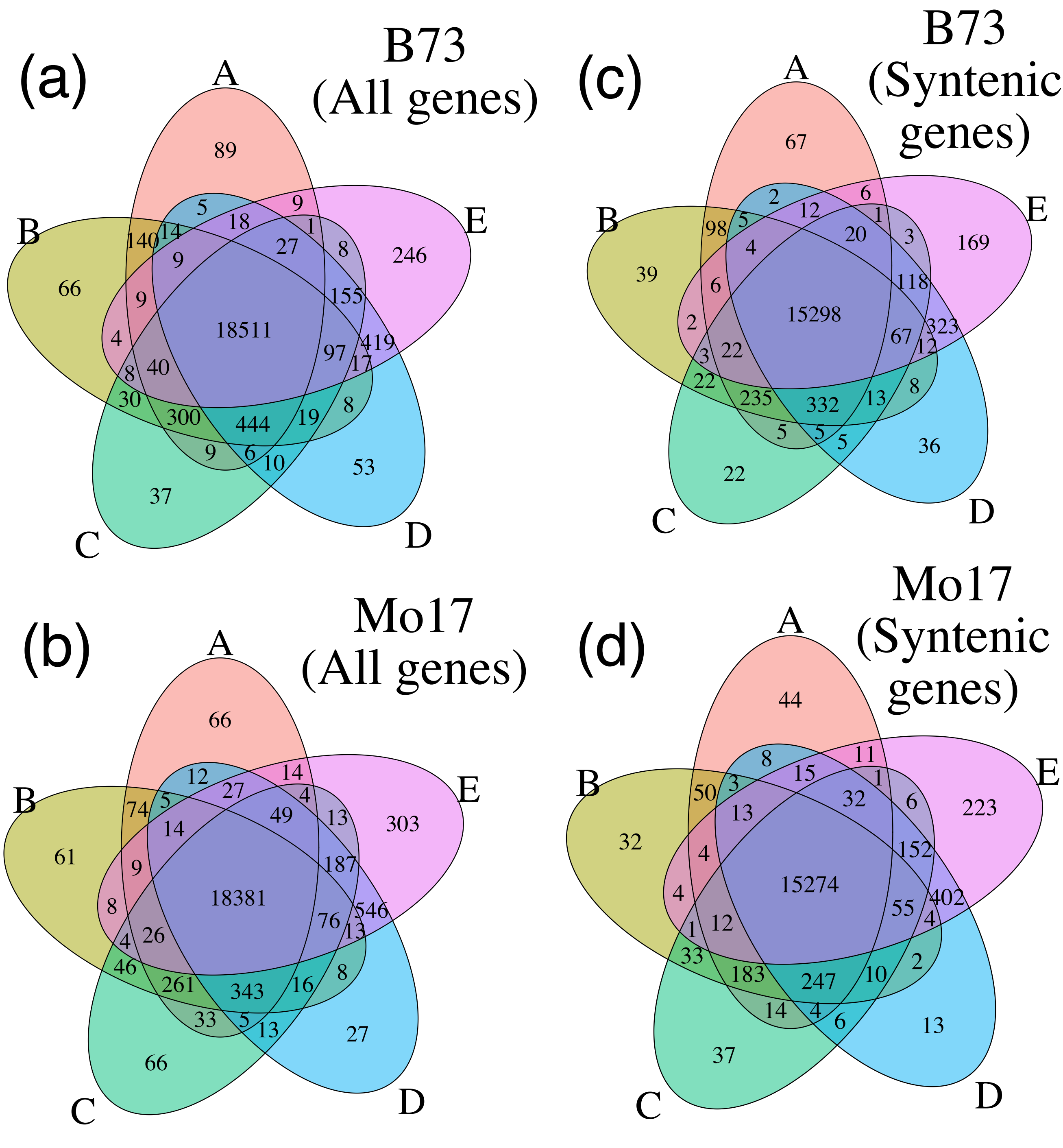

(a)
B73
(All genes)
A
B
C
D
E
89
5
9
14
140
18
1
27
8
246
66
9
9
155
18511
4
419
97
17
8
40
30
300
444
19
8
9
6
10
53
37
(b)
Mo17
(All genes)
A
B
C
D
E
66
12
14
74
5
27
4
13
303
61
14
49
9
187
18381
8
546
76
13
4
26
46
261
343
16
8
33
5
13
27
66
(c)
B73
(Syntenic
genes)
A
B
C
D
E
67
2
6
98
5
12
1
20
3
169
39
4
6
118
15298
2
323
67
12
3
22
22
235
332
13
8
5
5
5
36
22
(d)
Mo17
(Syntenic
genes)
A
B
C
D
E
44
8
11
50
3
15
1
32
6
223
32
13
4
152
15274
4
402
55
4
1
12
33
183
247
10
2
14
4
6
13
37
