# Supplementary material for: A multigenotype maize silk expression atlas reveals how exposure‐related stresses are mitigated following emergence from husk leaves
Source: Plant Genome. 2020 Oct 14;13(3):e20040. doi: 10.1002/tpg2.20040 (PMC12806928; doi:10.1002/tpg2.20040)
Supplement: Supplementary file 2 [file TPG2-13-e20040-s002.pdf]

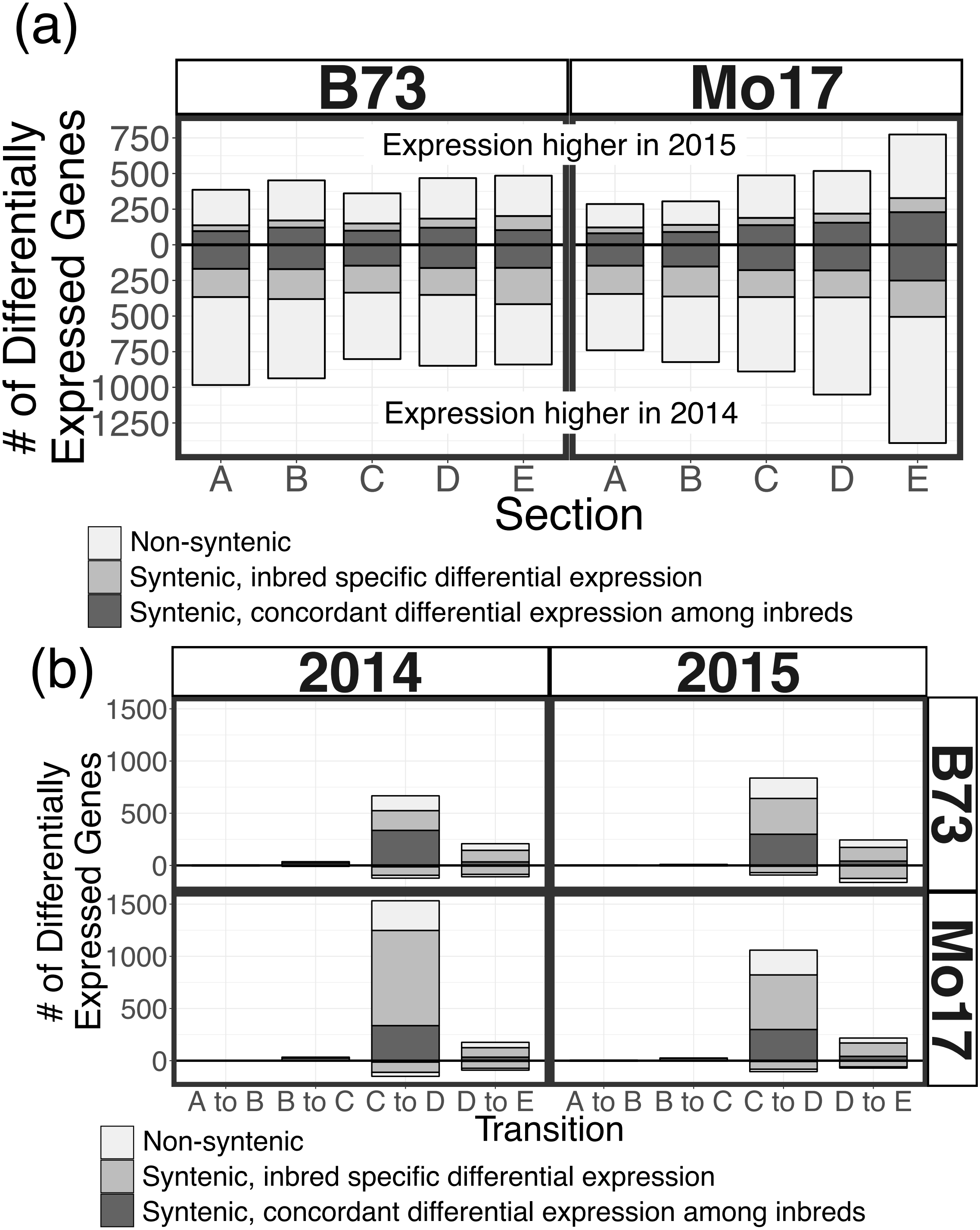

(a)
B73
Mo17
Expression higher in 2015
Expression higher in 2014
# of Differentially Expressed Genes
750
500
250
0
250
500
750
1000
1250
A
B
C
D
E
Section
Non-syntenic
Syntenic, inbred specific differential expression
Syntenic, concordant differential expression among inbreds
(b)
2014
2015
B73
Mo17
# of Differentially Expressed Genes
1500
1000
500
0
A to B
B to C
C to D
D to E
Transition
Non-syntenic
Syntenic, inbred specific differential expression
Syntenic, concordant differential expression among inbreds
