# Supplementary material for: A multigenotype maize silk expression atlas reveals how exposure‐related stresses are mitigated following emergence from husk leaves
Source: Plant Genome. 2020 Oct 14;13(3):e20040. doi: 10.1002/tpg2.20040 (PMC12806928; doi:10.1002/tpg2.20040)
Supplement: Supplementary file 3 [file TPG2-13-e20040-s003.pdf]

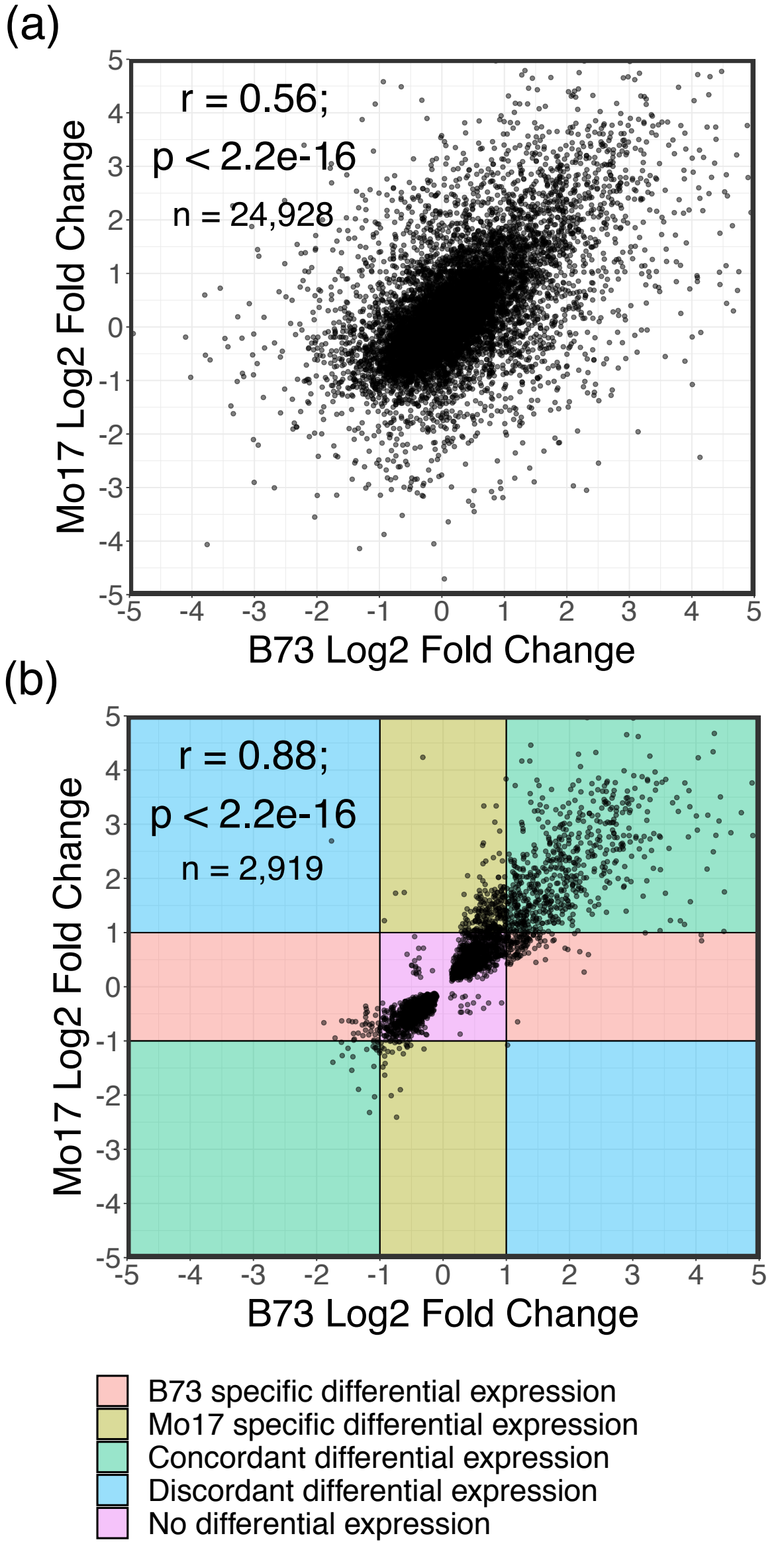
(a)
r = 0.56;
p < 2.2e-16
n = 24,928
Mo17 Log2 Fold Change
B73 Log2 Fold Change
(b)
r = 0.88;
p < 2.2e-16
n = 2,919
Mo17 Log2 Fold Change
B73 Log2 Fold Change
B73 specific differential expression
Mo17 specific differential expression
Concordant differential expression
Discordant differential expression
No differential expression
