# Supplementary material for: A multigenotype maize silk expression atlas reveals how exposure‐related stresses are mitigated following emergence from husk leaves
Source: Plant Genome. 2020 Oct 14;13(3):e20040. doi: 10.1002/tpg2.20040 (PMC12806928; doi:10.1002/tpg2.20040)
Supplement: Supplementary file 4 [file TPG2-13-e20040-s004.pdf]

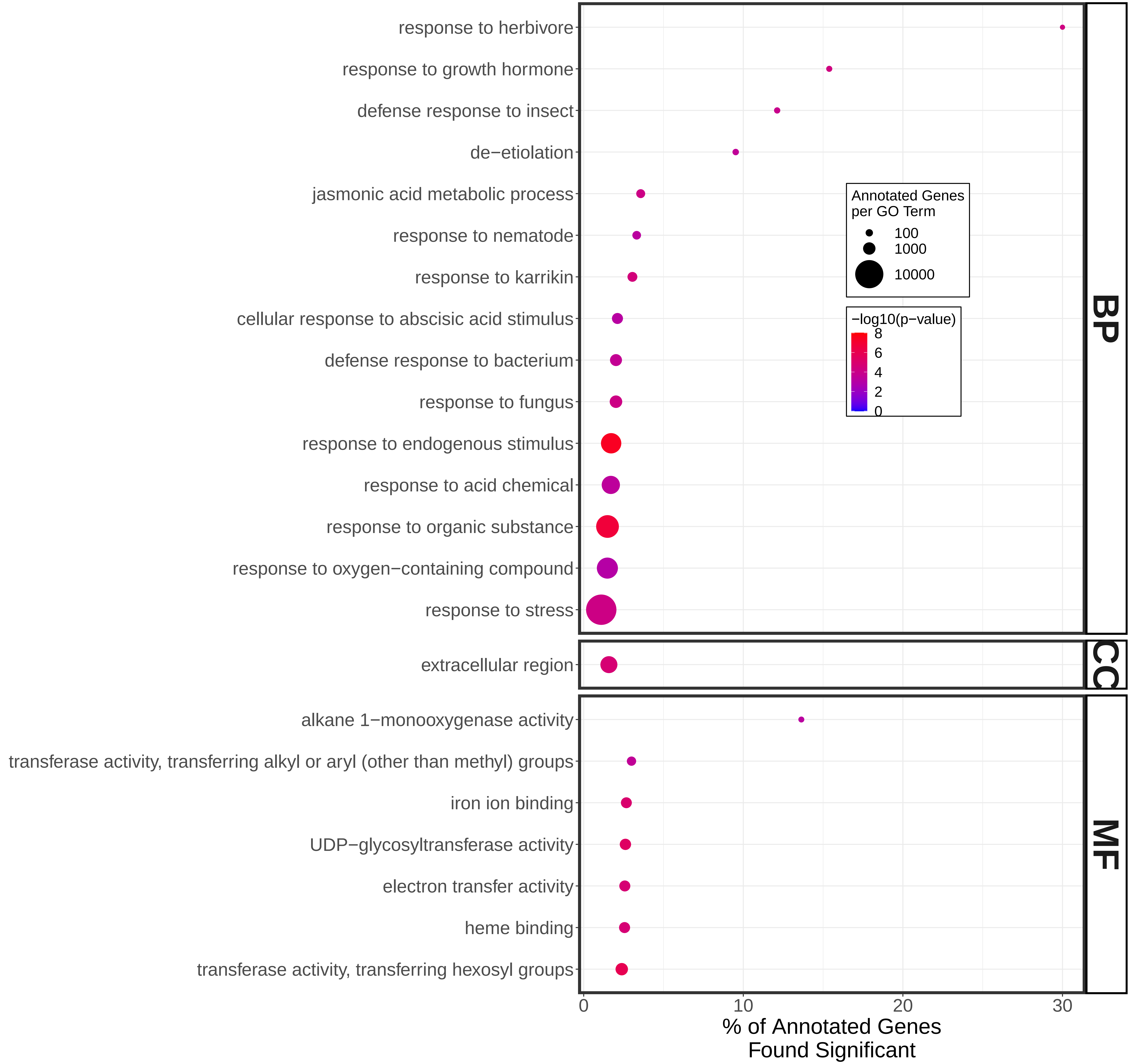
response to herbivore
response to growth hormone
defense response to insect
de−etiolation
jasmonic acid metabolic process
response to nematode
response to karrikin
cellular response to abscisic acid stimulus
defense response to bacterium
response to fungus
response to endogenous stimulus
response to acid chemical
response to organic substance
response to oxygen−containing compound
response to stress
extracellular region
alkane 1−monooxygenase activity
transferase activity, transferring alkyl or aryl (other than methyl) groups
iron ion binding
UDP−glycosyltransferase activity
electron transfer activity
heme binding
transferase activity, transferring hexosyl groups
BP
CC
MF
Annotated Genes per GO Term
100
1000
10000
−log10(p−value)
8
6
4
2
0
0
10
20
30
% of Annotated Genes Found Significant
