# Supplementary material for: A multigenotype maize silk expression atlas reveals how exposure‐related stresses are mitigated following emergence from husk leaves
Source: Plant Genome. 2020 Oct 14;13(3):e20040. doi: 10.1002/tpg2.20040 (PMC12806928; doi:10.1002/tpg2.20040)
Supplement: Supplementary file 5 [file TPG2-13-e20040-s005.pdf]

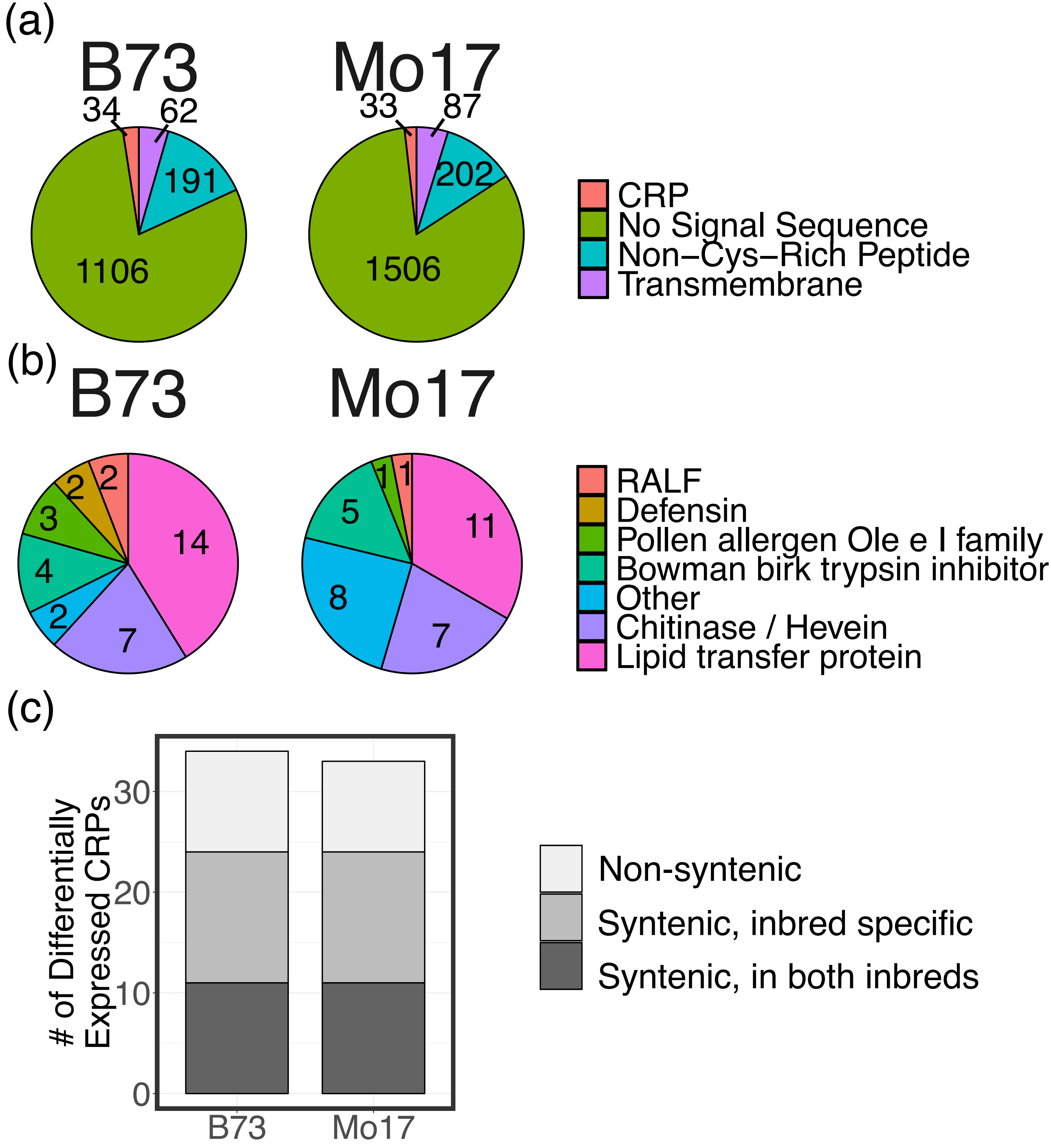
(a)
B73
Mo17
34
62
191
1106
33
87
202
1506
CRP
No Signal Sequence
Non−Cys−Rich Peptide
Transmembrane
(b)
B73
Mo17
2
2
3
4
2
7
14
1
1
5
8
7
11
RALF
Defensin
Pollen allergen Ole e I family
Bowman birk trypsin inhibitor
Other
Chitinase / Hevein
Lipid transfer protein
(c)
# of Differentially Expressed CRPs
0
10
20
30
B73
Mo17
Non-syntenic
Syntenic, inbred specific
Syntenic, in both inbreds
