# Supplementary material for: A multigenotype maize silk expression atlas reveals how exposure‐related stresses are mitigated following emergence from husk leaves
Source: Plant Genome. 2020 Oct 14;13(3):e20040. doi: 10.1002/tpg2.20040 (PMC12806928; doi:10.1002/tpg2.20040)
Supplement: Supplementary file 6 [file TPG2-13-e20040-s006.pdf]

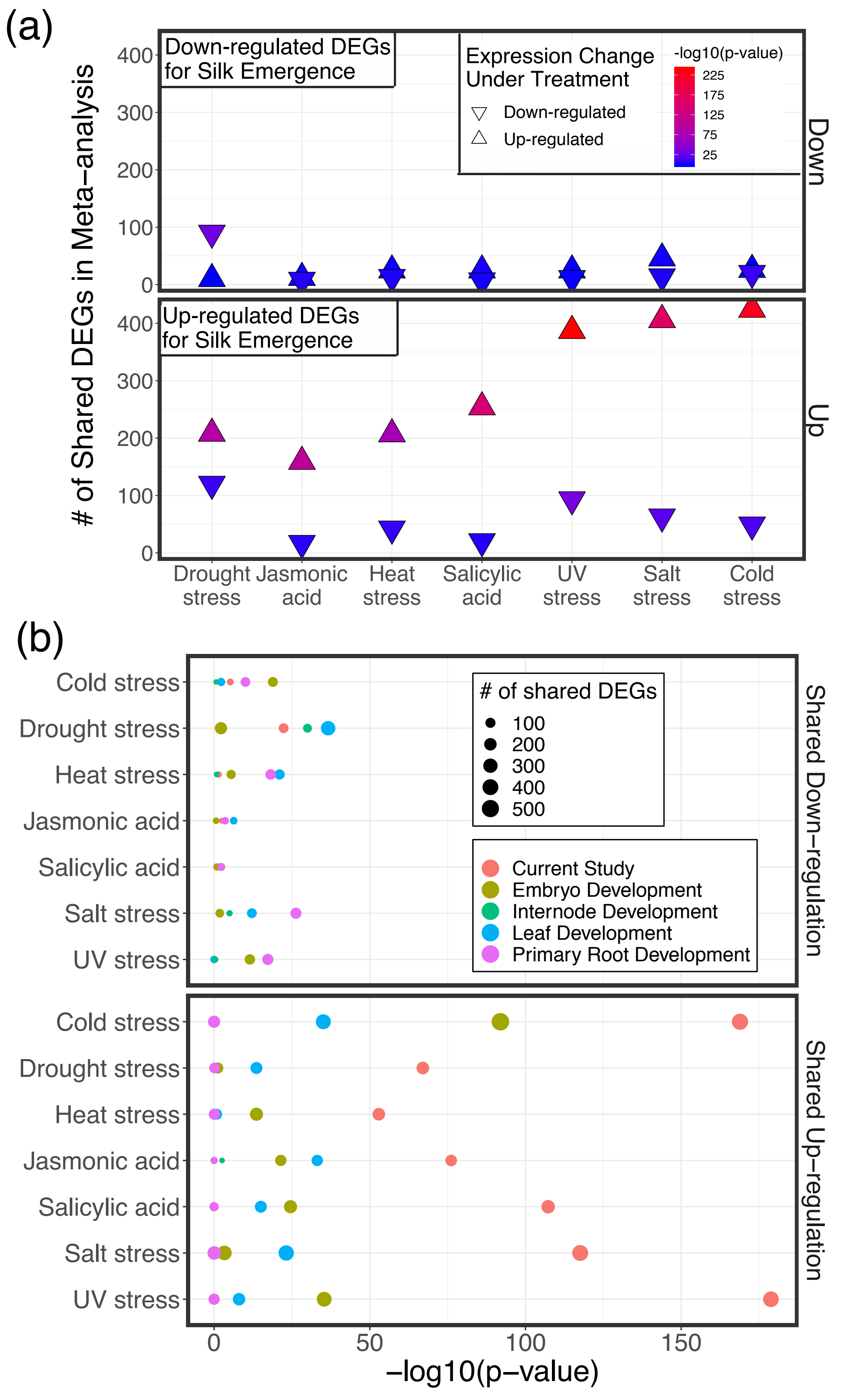
(a)
Down-regulated DEGs for Silk Emergence
Up-regulated DEGs for Silk Emergence
Expression Change Under Treatment
Down-regulated
Up-regulated
-log10(p-value)
225
175
125
75
25
# of Shared DEGs in Meta−analysis
400
300
200
100
0
Down
Up
Drought stress
Jasmonic acid
Heat stress
Salicylic acid
UV stress
Salt stress
Cold stress
(b)
# of shared DEGs
100
200
300
400
500
Current Study
Embryo Development
Internode Development
Leaf Development
Primary Root Development
Shared Down−regulation
Shared Up−regulation
0
50
100
150
−log10(p−value)
