# Supplementary material for: A multigenotype maize silk expression atlas reveals how exposure‐related stresses are mitigated following emergence from husk leaves
Source: Plant Genome. 2020 Oct 14;13(3):e20040. doi: 10.1002/tpg2.20040 (PMC12806928; doi:10.1002/tpg2.20040)
Supplement: Supplementary file 7 [file TPG2-13-e20040-s007.pdf]

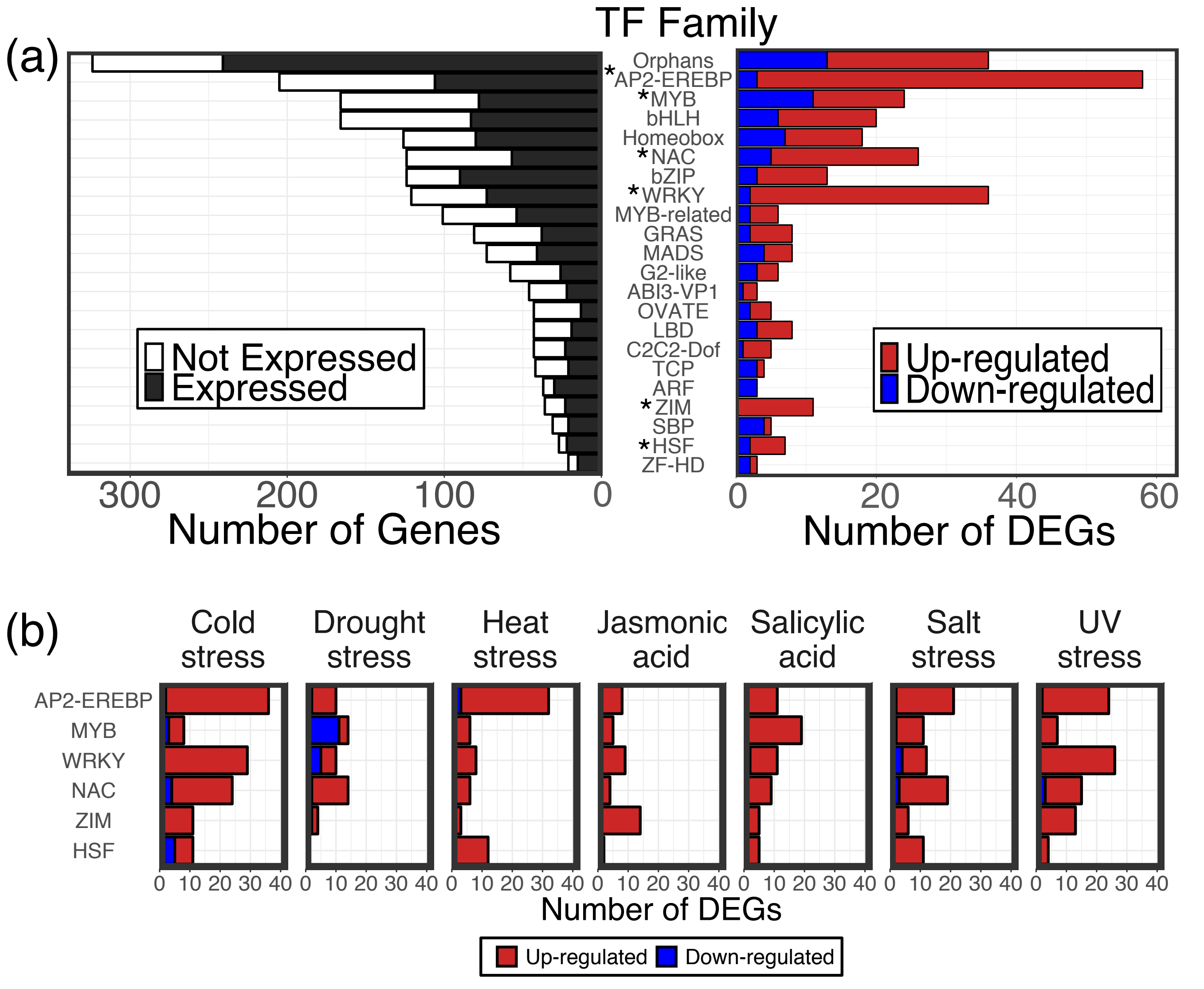

(a)
TF Family
Orphans
*AP2-EREBP
*MYB
bHLH
Homeobox
*NAC
bZIP
*WRKY
MYB-related
GRAS
MADS
G2-like
ABI3-VP1
OVATE
LBD
C2C2-Dof
TCP
ARF
*ZIM
SBP
*HSF
ZF-HD
Not Expressed
Expressed
300 200 100 0
Number of Genes
Up-regulated
Down-regulated
0 20 40 60
Number of DEGs
(b)
Cold stress
Drought stress
Heat stress
Jasmonic acid
Salicylic acid
Salt stress
UV stress
AP2-EREBP
MYB
WRKY
NAC
ZIM
HSF
0 10 20 30 40
Number of DEGs
Up-regulated
Down-regulated
