# Supplementary material for: A multigenotype maize silk expression atlas reveals how exposure‐related stresses are mitigated following emergence from husk leaves
Source: Plant Genome. 2020 Oct 14;13(3):e20040. doi: 10.1002/tpg2.20040 (PMC12806928; doi:10.1002/tpg2.20040)
Supplement: Supplementary file 8 [file TPG2-13-e20040-s008.pdf]

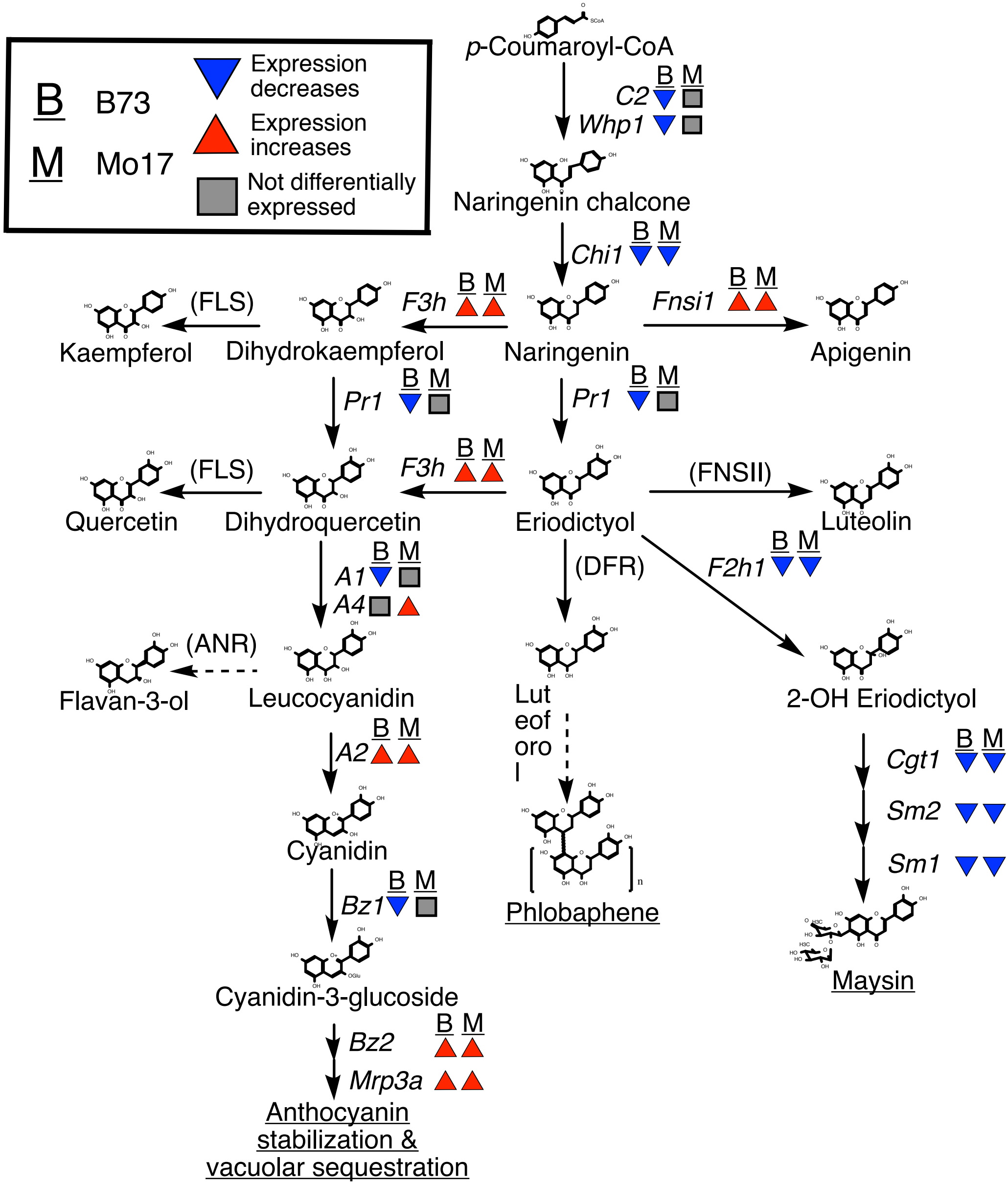
B B73
M Mo17
Expression decreases
Expression increases
Not differentially expressed
p-Coumaroyl-CoA
C2
Whp1
Naringenin chalcone
Chi1
Naringenin
F3h
Fnsi1
Dihydrokaempferol
(FLS)
Kaempferol
Apigenin
Pr1
Eriodictyol
Dihydroquercetin
Quercetin
(FNSII)
Luteolin
F2h1
A1
A4
(DFR)
(ANR)
Flavan-3-ol
Leucocyanidin
Luteoforol
2-OH Eriodictyol
A2
Cyanidin
Cgt1
Sm2
Sm1
Bz1
Phlobaphene
Maysin
Cyanidin-3-glucoside
Bz2
Mrp3a
Anthocyanin stabilization & vacuolar sequestration
