# Supplementary material for: A multigenotype maize silk expression atlas reveals how exposure‐related stresses are mitigated following emergence from husk leaves
Source: Plant Genome. 2020 Oct 14;13(3):e20040. doi: 10.1002/tpg2.20040 (PMC12806928; doi:10.1002/tpg2.20040)
Supplement: Supplementary file 9 [file TPG2-13-e20040-s009.pdf]

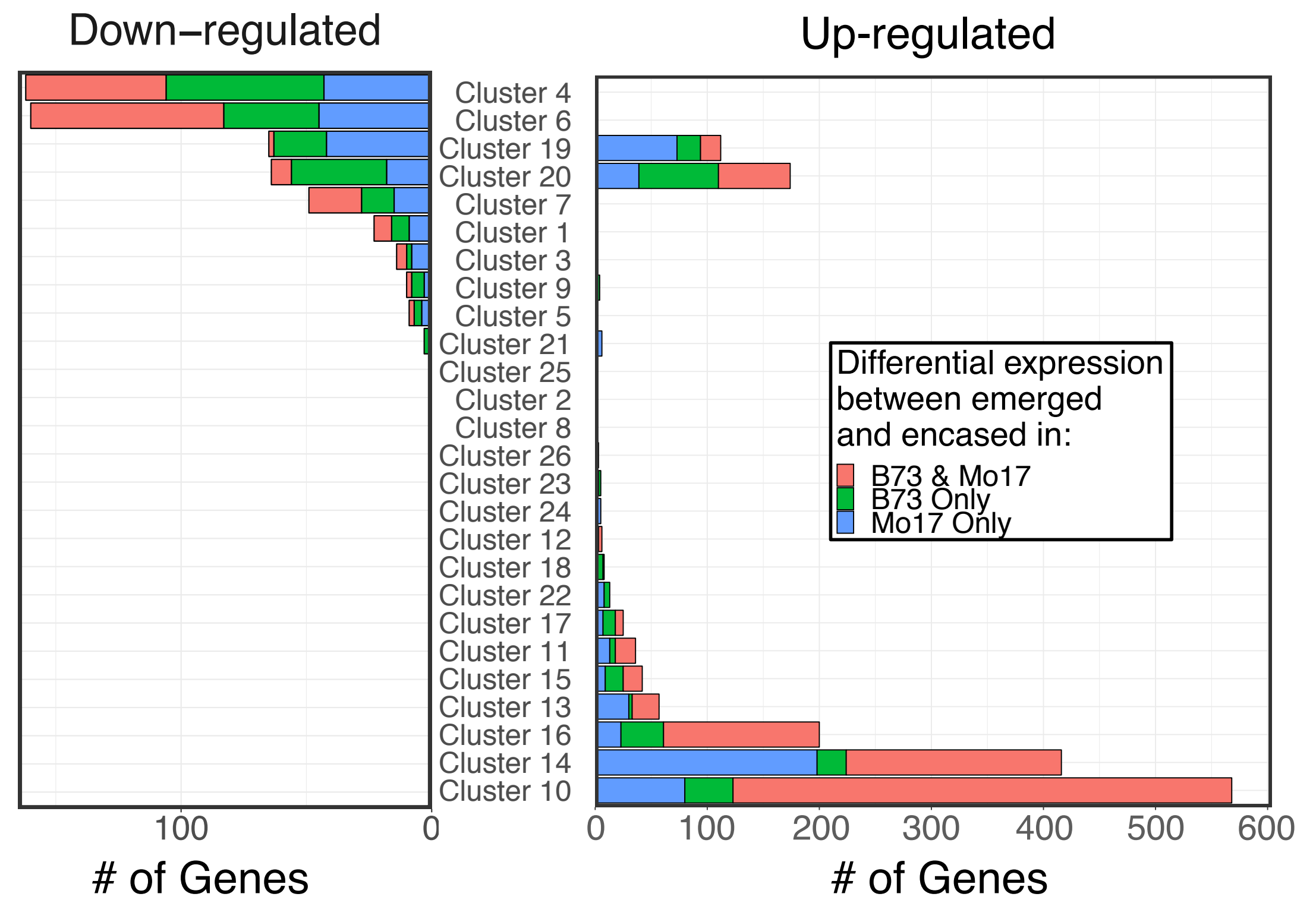

Down–regulated
Up-regulated
Cluster 4
Cluster 6
Cluster 19
Cluster 20
Cluster 7
Cluster 1
Cluster 3
Cluster 9
Cluster 5
Cluster 21
Cluster 25
Cluster 2
Cluster 8
Cluster 26
Cluster 23
Cluster 24
Cluster 12
Cluster 18
Cluster 22
Cluster 17
Cluster 11
Cluster 15
Cluster 13
Cluster 16
Cluster 14
Cluster 10
Differential expression between emerged and encased in:
B73 & Mo17
B73 Only
Mo17 Only
100
0
0
100
200
300
400
500
600
# of Genes
# of Genes
