# Supplementary material for: A multigenotype maize silk expression atlas reveals how exposure‐related stresses are mitigated following emergence from husk leaves
Source: Plant Genome. 2020 Oct 14;13(3):e20040. doi: 10.1002/tpg2.20040 (PMC12806928; doi:10.1002/tpg2.20040)
Supplement: Supplementary file 10 [file TPG2-13-e20040-s019.pdf]

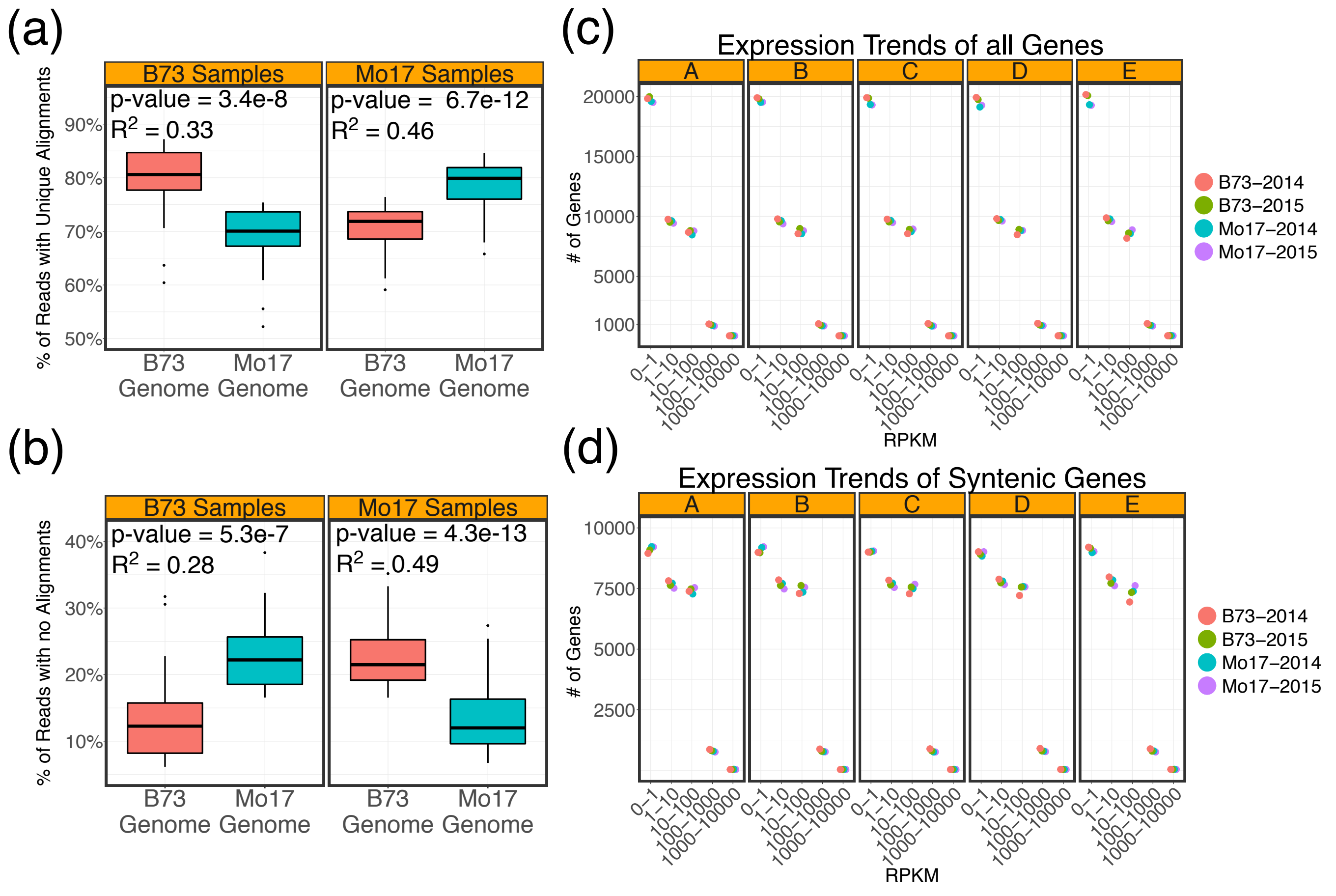

(a)
B73 Samples
Mo17 Samples
p-value = 3.4e-8
R2 = 0.33
p-value = 6.7e-12
R2 = 0.46
% of Reads with Unique Alignments
90%
80%
70%
60%
50%
B73 Genome
Mo17 Genome
(b)
B73 Samples
Mo17 Samples
p-value = 5.3e-7
R2 = 0.28
p-value = 4.3e-13
R2 = 0.49
% of Reads with no Alignments
40%
30%
20%
10%
(c)
Expression Trends of all Genes
A
B
C
D
E
# of Genes
20000
15000
10000
5000
1000
0–1
1–10
10–100
100–1000
1000–10000
RPKM
B73–2014
B73–2015
Mo17–2014
Mo17–2015
(d)
Expression Trends of Syntenic Genes
10000
7500
5000
2500
