# Supplementary material for: A multigenotype maize silk expression atlas reveals how exposure‐related stresses are mitigated following emergence from husk leaves
Source: Plant Genome. 2020 Oct 14;13(3):e20040. doi: 10.1002/tpg2.20040 (PMC12806928; doi:10.1002/tpg2.20040)
Supplement: Supplementary file 11 [file TPG2-13-e20040-s020.pdf]

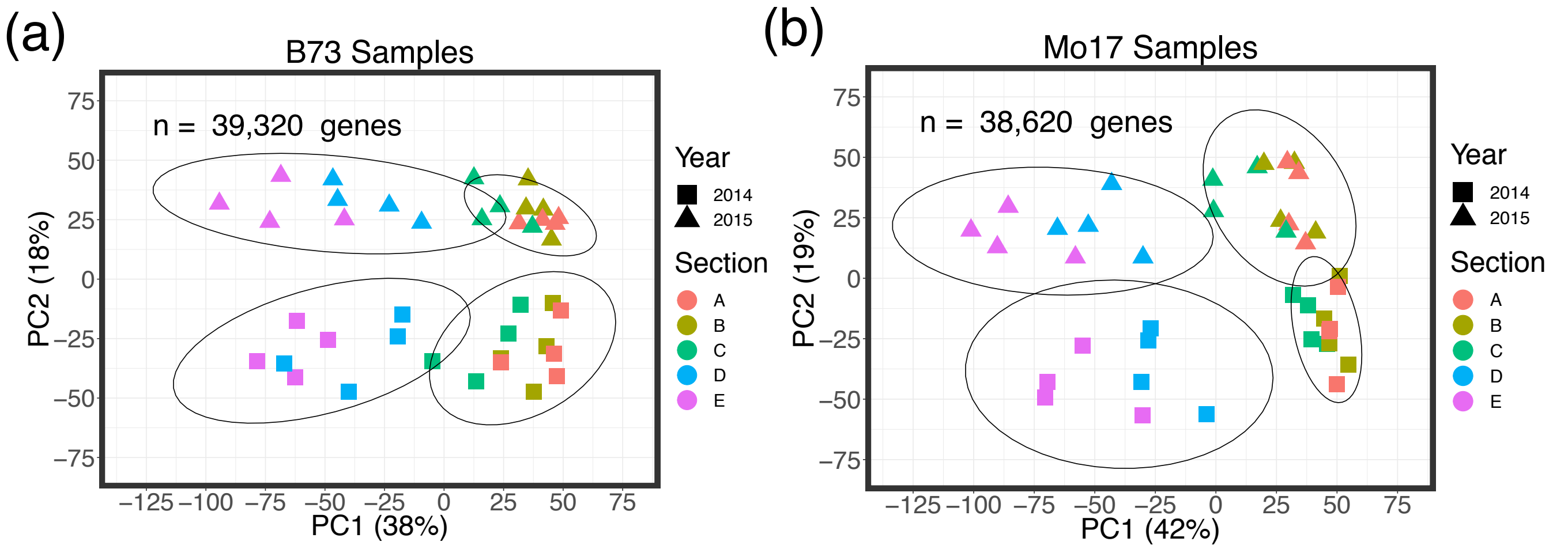

(a)
B73 Samples
n = 39,320 genes
PC2 (18%)
PC1 (38%)
75
50
25
0
−25
−50
−75
−125
−100
−75
−50
−25
0
25
50
75
Year
2014
2015
Section
A
B
C
D
E
(b)
Mo17 Samples
n = 38,620 genes
PC2 (19%)
PC1 (42%)
