# Supplementary material for: A multigenotype maize silk expression atlas reveals how exposure‐related stresses are mitigated following emergence from husk leaves
Source: Plant Genome. 2020 Oct 14;13(3):e20040. doi: 10.1002/tpg2.20040 (PMC12806928; doi:10.1002/tpg2.20040)
Supplement: Supplementary file 12 [file TPG2-13-e20040-s021.pdf]

(a) All genes

Transition				B73 genes		Mo17 genes	
A to B	B to C	C to D	D to E	Number	Proportion	Number	Proportion
No Change	No Change	No Change	No Change	37529	0.954	36589	0.947
No Change	No Change	Increases	No Change	968	0.025	1324	0.034
No Change	No Change	No Change	Decreases	222	0.006	121	0.003
No Change	No Change	Increases	Increases	197	0.005	246	0.006
No Change	No Change	No Change	Increases	172	0.004	77	0.002
No Change	No Change	Decreases	No Change	111	0.003	171	0.004
No Change	Increases	Increases	No Change	49	0.001	39	0.001
No Change	No Change	Decreases	Decreases	45	0.001	20	0.001
No Change	Increases	Increases	Increases	17	< 0.001	25	0.001
No Change	No Change	Increases	Decreases	3	< 0.001	0	0
No Change	Increases	No Change	No Change	2	< 0.001	2	< 0.001
No Change	No Change	Decreases	Increases	1	< 0.001	2	< 0.001
No Change	Decreases	Decreases	No Change	1	< 0.001	1	< 0.001
Increases	No Change	No Change	No Change	1	< 0.001	1	< 0.001
No Change	Decreases	No Change	No Change	1	< 0.001	0	0
Increases	Increases	Increases	Increases	1	< 0.001	0	0
Decreases	No Change	No Change	No Change	0	0	1	< 0.001
Decreases	Increases	No Change	No Change	0	0	1	< 0.001

(b) Syntenic genes

Transition				B73 genes		Mo17 genes	
A to B	B to C	C to D	D to E	Number	Proportion	Number	Proportion
No Change	No Change	No Change	No Change	23544	0.944	23305	0.935
No Change	No Change	Increases	No Change	767	0.031	1057	0.042
No Change	No Change	No Change	Decreases	172	0.007	103	0.004
No Change	No Change	Increases	Increases	142	0.006	191	0.008
No Change	No Change	No Change	Increases	122	0.005	61	0.002
No Change	No Change	Decreases	No Change	90	0.004	130	0.005
No Change	No Change	Decreases	Decreases	39	0.002	19	0.001
No Change	Increases	Increases	No Change	33	0.001	33	0.001
No Change	Increases	Increases	Increases	13	0.001	18	0.001
No Change	No Change	Increases	Decreases	3	< 0.001	0	0
No Change	Increases	No Change	No Change	1	< 0.001	2	< 0.001
No Change	Decreases	Decreases	No Change	1	< 0.001	1	< 0.001
Increases	Increases	Increases	Increases	1	< 0.001	0	0
Increases	No Change	No Change	No Change	0	0	1	< 0.001
Decreases	Increases	No Change	No Change	0	0	1	< 0.001
No Change	No Change	Decreases	Increases	0	0	1	< 0.001

No Change | Expression Increases | Expression Decreases
