# Supplementary material for: A multigenotype maize silk expression atlas reveals how exposure‐related stresses are mitigated following emergence from husk leaves
Source: Plant Genome. 2020 Oct 14;13(3):e20040. doi: 10.1002/tpg2.20040 (PMC12806928; doi:10.1002/tpg2.20040)
Supplement: Supplementary file 13 [file TPG2-13-e20040-s012.docx]

**Table S3.** References and DOIs of previous transcriptomic studies of stress treated seedlings and developmentally-related controls used in a meta-analysis in the current

| Subject | Reference | DOI |
| --- | --- | --- |
| Cold stress | Makarevitch et al., 2015 | 10.1371/journal.pgen.1004915 |
| Drought stress | Zhang et al., 2018 | 10.1186/s12870-018-1281-x |
| Embryo development | Walley et al., 2016 | 10.1126/science.aag1125 |
| Heat stress | Makarevitch et al., 2015 | 10.1371/journal.pgen.1004915 |
| Internode development | Walley et al., 2016 | 10.1126/science.aag1125 |
| Jasmonic acid | Wang et al., 2017 | 10.1371/journal.pone.0177739 |
| Leaf development | Li et al., 2010 | 10.1038/ng.703 |
| Primary root development | Walley et al., 2016 | 10.1126/science.aag1125 |
| Salicylic acid | Ziemann et al., 2018 | 10.1038/s41477-018-0116-y |
| Salt stress | Makarevitch et al., 2015 | 10.1371/journal.pgen.1004915 |
| UV stress | Makarevitch et al., 2015 | 10.1371/journal.pgen.1004915 |

investigation.
