# Supplementary material for: A multigenotype maize silk expression atlas reveals how exposure‐related stresses are mitigated following emergence from husk leaves
Source: Plant Genome. 2020 Oct 14;13(3):e20040. doi: 10.1002/tpg2.20040 (PMC12806928; doi:10.1002/tpg2.20040)
Supplement: Supplementary file 14 [file TPG2-13-e20040-s013.docx]

| Stress Treatment | Treatment Up-regulated DEGs | Treatment Down-regulated DEGs | Treatment Up-regulated DEGs Expressed in Silks | Treatment Down-regulated DEGs Expressed in Silks |
| --- | --- | --- | --- | --- |
| Cold stress | 1,754 | 340 | 1,435 | 265 |
| Drought stress | 1,005 | 1,494 | 895 | 1,120 |
| Heat stress | 1,200 | 401 | 899 | 320 |
| Jasmonic acid | 524 | 132 | 372 | 77 |
| Salicylic acid | 967 | 220 | 734 | 149 |
| Salt stress | 2,202 | 419 | 1,769 | 313 |
| UV stress | 1,373 | 534 | 966 | 448 |

**Table S4.** Numbers of total and silk expressed down- and up-regulated DEGs identified in previous transcriptomic studies of stress treated seedlings used for a meta-analysis in the current investigation (See Table S3).
